# Phosphorylation of the novel mTOR substrate Unkempt regulates cellular morphogenesis

**DOI:** 10.1016/j.jbc.2022.102788

**Published:** 2022-12-09

**Authors:** Pranetha Baskaran, Simeon R. Mihaylov, Elin Vinsland, Kriti Shah, Lucy Granat, Sila K. Ultanir, Andrew R. Tee, Jernej Murn, Joseph M. Bateman

**Affiliations:** 1 Maurice Wohl Clinical Neuroscience Institute, King’s College London, London, UK; 2 Kinases and Brain Development Lab, The Francis Crick Institute, London, UK; 3 Division of Molecular Neurobiology, Department of Medical Biochemistry and Biophysics, Karolinska Institute, Stockholm, Sweden; 4 Department of Biochemistry, University of California, Riverside, California, USA; 5 Cancer and Genetics Building, Division of Cancer and Genetics, School of Medicine, Cardiff University, Heath Park Way, Cardiff, UK

**Keywords:** Unkempt, mTOR, cellular morphogenesis, Raptor, intrinsically disordered region, phosphorylation, CLIP, crosslinking and immunoprecipitation, DOX, doxycycline, HA, hemagglutinin, IDR, intrinsically disordered region, MS, mass spectrometry, mTOR, mechanistic target of rapamycin, mTORC1, mTOR complex 1, mTORC2, mTOR complex 2, PPI, protein–protein interaction, P-Unk, Unkempt phospho-specific antibody, rpS6, ribosomal protein S6, ZF, zinc finger

## Abstract

Mechanistic target of rapamycin (mTOR) is a protein kinase that integrates multiple inputs to regulate anabolic cellular processes. For example, mTOR complex 1 (mTORC1) has key functions in growth control, autophagy, and metabolism. However, much less is known about the signaling components that act downstream of mTORC1 to regulate cellular morphogenesis. Here, we show that the RNA-binding protein Unkempt, a key regulator of cellular morphogenesis, is a novel substrate of mTORC1. We show that Unkempt phosphorylation is regulated by nutrient levels and growth factors *via* mTORC1. To analyze Unkempt phosphorylation, we immunoprecipitated Unkempt from cells in the presence or the absence of the mTORC1 inhibitor rapamycin and used mass spectrometry to identify mTORC1-dependent phosphorylated residues. This analysis showed that mTORC1-dependent phosphorylation is concentrated in a serine-rich intrinsically disordered region in the C-terminal half of Unkempt. We also found that Unkempt physically interacts with and is directly phosphorylated by mTORC1 through binding to the regulatory-associated protein of mTOR, Raptor. Furthermore, analysis in the developing brain of mice lacking TSC1 expression showed that phosphorylation of Unkempt is mTORC1 dependent *in vivo*. Finally, mutation analysis of key serine/threonine residues in the serine-rich region indicates that phosphorylation inhibits the ability of Unkempt to induce a bipolar morphology. Phosphorylation within this serine-rich region thus profoundly affects the ability of Unkempt to regulate cellular morphogenesis. Taken together, our findings reveal a novel molecular link between mTORC1 signaling and cellular morphogenesis.

Mechanistic target of rapamycin (mTOR) is a large serine/threonine kinase that forms the catalytic subunit of two complexes, mTOR complex 1 (mTORC1) and mTOR complex 2 (mTORC2) (1). mTORC1 integrates signaling inputs from cellular nutrients, growth factors, energy, and stress to regulate a range of anabolic processes. Hyperactivation of mTORC1 signaling is implicated in myriad disease contexts, including cancer, metabolic, immune, and neurological diseases (2, 3). mTORC2 was first shown to regulate the actin cytoskeleton *via* the AGC kinase PKCα and was subsequently shown to have additional roles in cell proliferation and survival (1, 4, 5).

mTOR regulates the phosphorylation of several hundred proteins, dependent on the cell type (6, 7, 8, 9, 10). Relatively few direct mTOR substrates have however been characterized in detail. Well-characterized mTORC1 substrates include S6K, 4E-BP1, and the adaptor protein Grb10, all regulators of cell growth and the protein kinase ULK1, which regulates autophagosome biogenesis (1, 6, 7, 11, 12). mTORC2 phosphorylates AKT and SGK1 to control cell proliferation and survival (5, 13, 14). mTORC2 also phosphorylates multiple PKC isoforms, including PKCα, PKBβII, and PKCζ, to regulate actin cytoskeletal dynamics (15, 16, 17).

In contrast to mTORC2, the role of mTORC1 in cellular morphogenesis is much less well understood. We previously showed that the zinc finger (ZF)/RING domain protein Unkempt acts genetically downstream of mTOR in the developing retina and central nervous system in *Drosophila* (18, 19, 20, 21). Mammalian Unkempt is a key regulator of cell morphogenesis and, through its ZF domain, binds mRNAs involved in control of protein synthesis and cell shape to affect their translation and to induce the establishment of the early neuronal morphology (22, 23).

Here, we show that mammalian Unkempt is abundantly phosphorylated and that phosphorylation is acutely sensitive to mTORC1 activity. Unkempt physically interacts with mTORC1 by binding to regulatory-associated protein of mTOR (Raptor), which mediates direct phosphorylation by mTOR at multiple serine/threonine residues in the highly serine-rich intrinsically disordered region (IDR). Using mice with conditional knockout of *Tsc1* to activate mTORC1 in the nervous system, we find that Unkempt phosphorylation is mTORC1 dependent *in vivo*. Finally, we show that phosphorylation in the serine-rich region suppresses the capacity of Unkempt to induce a bipolar morphology, a critical cellular activity of Unkempt.

## Results

### mTORC1 activity regulates Unkempt phosphorylation

Unkempt is a highly conserved RNA-binding protein that plays critical roles in controlling cell proliferation and differentiation in *Drosophila* and cellular morphogenesis in mammals (18, 21, 23, 24). How the activity of Unkempt might be regulated remains unknown. Recent studies have suggested that Unkempt acts genetically downstream of mTORC1 (18, 21, 24), but it is unclear whether mTORC1 signaling might be linked to the function of Unkempt. Mammalian Unkempt is either not expressed or expressed at very low levels in non-neural cell lines but is abundant in cells of neuronal lineages (23). We therefore used human neuroblastoma SH-SY5Y cells to determine whether Unkempt is regulated in an mTORC1-dependent manner.

**Figure 1:**
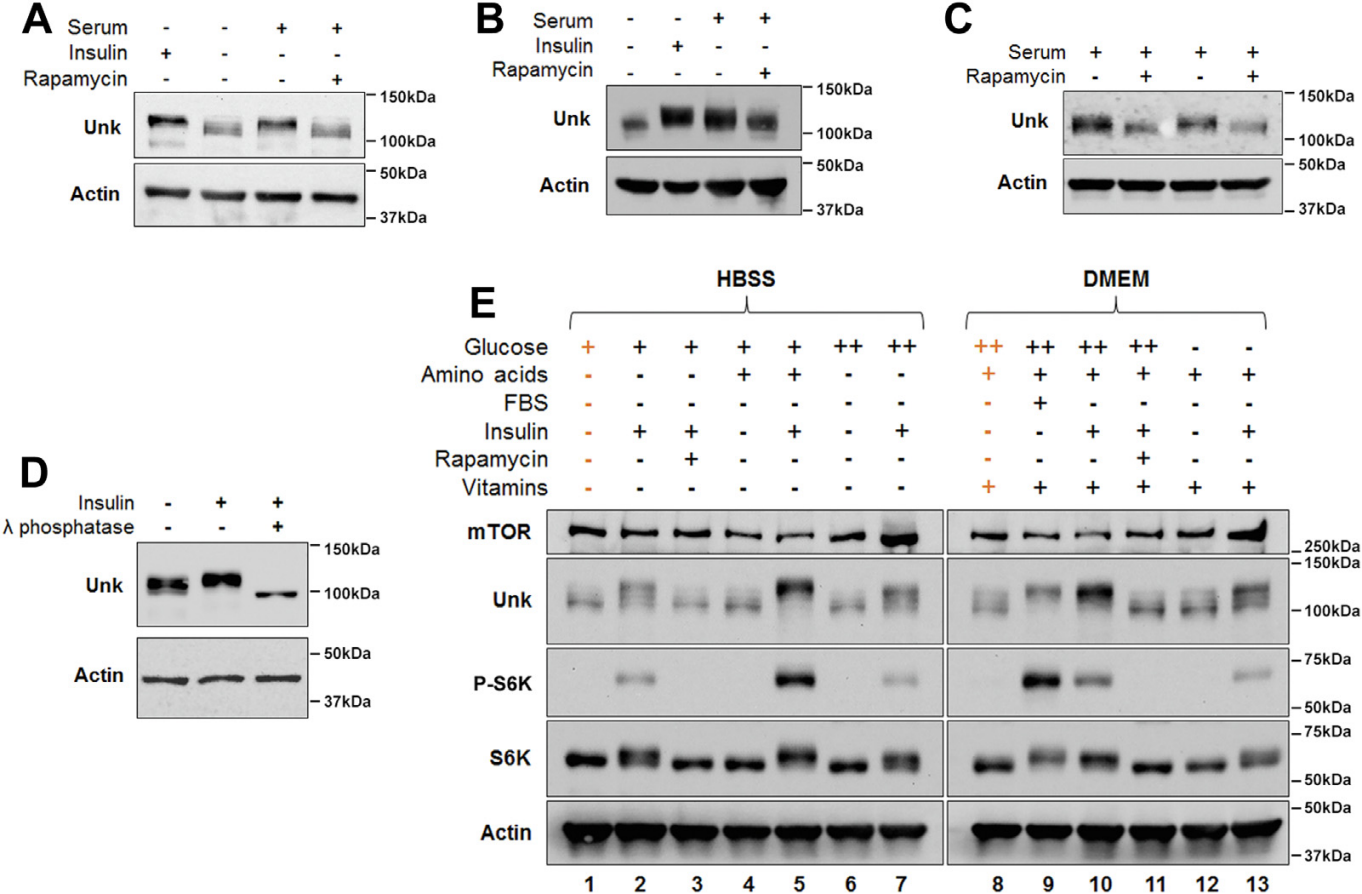
**Phosphorylation of Unkempt is mTORC1 activity dependent.***A*, Western blot showing serum starvation followed by insulin stimulation of SH-SY5Y cells increases, whereas rapamycin treatment decreases the electrophoretic mobility of Unkempt. *B* and *C*, rapamycin treatment decreases the electrophoretic mobility of Unkempt in mouse N2a cells and mouse E14.5 primary cortical cells. *D*, treatment of SH-SY5Y cell lysate with lambda protein phosphatase abrogates the electrophoretic mobility shift in Unkempt. *E*, Unkempt phosphorylation is highly sensitive to nutrient levels. SH-SY5Y cells were grown in minimal media lacking vitamins and amino acids (HBSS) or DMEM. Additional nutrients were added as indicated. *Orange columns* indicate control for that panel. SH-SY5Y and N2a cells were grown in DMEM + 10% fetal bovine serum (FBS), then serum starved for 16 h in DMEM alone, then incubated for 1 h in DMEM ± 1 μM insulin, or DMEM + 10% FBS ± 1 μM rapamycin. C57BL/6J mouse primary cortical cells were grown in Neurobasal medium. For the experiment in (*E*), SH-SY5Y cells were grown in DMEM + 10% FBS, then serum starved for 16 h in DMEM alone, and then incubated for 2.5 h in HBSS medium (which contains 1 g/l of glucose and lacks amino acids and vitamins). Trophic factors were then added to the starved cells for 25 min with a combination of glucose (+, 1 g/l; ++, 4.5 g/l), amino acids (l-alanyl-l-glutamine 0.868 g/l, l-arginine 0.084 g/l, l-cystine 0.0626 g/l, glycine 0.03 g/l, l-histidine 0.042 g/l, l-isoleucine 0.105 g/l, l-leucine 0.105 g/l, l-lysine 0.146 g/l, l-methionine 0.03 g/l, l-phenylalanine 0.066 g/l, l-serine 0.042 g/l, l-threonine 0.095 g/l, l-tryptophan 0.016 g/l, l-tyrosine 0.104 g/l, and l-valine 0.094 g/l), FBS (10%), insulin (1 μM), or rapamycin (1 μM) in HBSS or glucose-free DMEM. DMEM, Dulbecco’s modified Eagle’s medium; HBSS, Hank’s balanced salt solution; mTORC1, mechanistic target of rapamycin complex 1.

In serum-starved neuroblastoma SH-SY5Y cells, we found that stimulation of the mTORC1 pathway with insulin or serum readdition caused a mobility shift of Unkempt to a higher resolved band, whereas direct inhibition of mTORC1 kinase activity with rapamycin prevented this shift (Fig. 1*A*). A strict correlation between inhibition of mTORC1, as judged by phosphorylation of ribosomal protein S6 (rpS6), and decreased mobility of Unkempt was observed over a wide range of rapamycin concentrations (Fig. S1*A*). Moreover, a phospho-specific antibody against Unkempt (see details later and Fig. 3*D*) detected reduced levels of phosphorylated Unkempt in rapamycin-treated cells (Fig. S1*A*). Rapamycin treatment also caused increased phosphorylation of AKT at Ser473 as a result of the established feedback loop between S6K and the insulin receptor substrate (25, 26) (Fig. S1*A*).

Unkempt showed a similar mTORC1-dependent mobility shift in response to rapamycin in mouse Neuro-2a neuroblastoma cells (Fig. 1*B*) and in mouse E14.5 primary cortical cells (Fig. 1*C*). After insulin stimulation, lambda protein phosphatase treatment of the SH-SY5Y cell lysate converted a higher resolving Unkempt band to a lower resolving species, below that in unstimulated cells (Fig. 1*D*). Together, these data suggest that Unkempt is phosphorylated in an mTORC1-dependent manner and exhibits a basal level of phosphorylation in unstimulated cells.

mTORC1 can be regulated by multiple signaling inputs, including insulin, amino acids, and ATP levels (1). We found that addition of amino acids alone in the media did not suffice to activate mTORC1, as judged by phosphorylation of S6K, or increase Unkempt phosphorylation (Fig. 1*E*, compare *lanes* 1 and 4). However, the presence of amino acids amplified the effect of insulin stimulation, as seen both by increased mTORC1 activity and enhanced phosphorylation of Unkempt (Fig. 1*E*, compare *lanes* 2 and 5). Similarly, increased glucose concentration, while having little effect on its own (Fig. 1*E*, compare *lanes* 8 and 12), potentiated mTORC1 activity and the phosphorylation of Unkempt primed by insulin stimulation in the presence of amino acids (Fig. 1*E*, compare *lanes* 10 and 13). The effects of amino acids and glucose were mTORC1 dependent, as rapamycin prevented the phosphorylation of Unkempt in the presence of amino acids, glucose, and insulin together (Fig. 1*E*, compare *lanes* 10 and 11). Unkempt phosphorylation is therefore acutely sensitive to nutrients and growth factors that regulate mTORC1.

**Figure 2:**
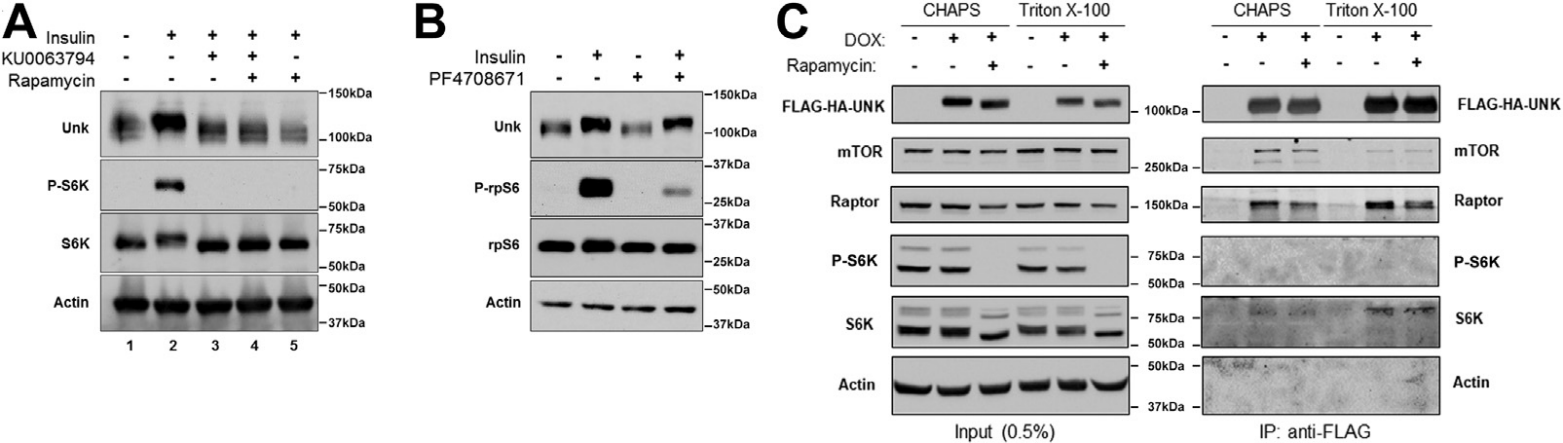
**Unkempt phosphorylation is regulated by mTORC1 and Unkempt physically interacts with the mTORC1.***A*, treatment of SH-SY5Y cells with KU0063794 alone, rapamycin alone, or both KU0063794 and rapamycin prevent the increase in Unkempt phosphorylation caused by insulin stimulation. *B*, inhibition of S6K with PF4708671 does not prevent the phosphorylation of Unkempt. *C*, Unkempt physically interacts with Raptor and mTOR. Inducible HeLa S3 cells were treated with 1 μg/ml doxycycline for 24 h to induce the expression of FLAG-HA-Unkempt. Extract from noninduced HeLa S3 cells (DOX−) was used as a negative control. Both SH-SY5Y (*A* and *B*) and HeLa S3 cells (*C*) were grown in DMEM + 10% FBS, then serum-starved for 16 h in DMEM alone, then incubated for 1 h in DMEM containing 1 μM insulin, 1 μM rapamycin, 1 μM KU0063794, or 10 μM PF4708671, as indicated. DMEM, Dulbecco’s modified Eagle’s medium; FBS, fetal bovine serum; HA, hemagglutinin; mTOR, mechanistic target of rapamycin; mTORC1, mTOR complex 1.

mTOR is a component of both mTORC1 and mTORC2. Prolonged rapamycin treatment can inhibit mTORC2 (13), but short-term (1 h) rapamycin treatment of SH-SY5Y cells did not inhibit mTORC2, as judged by AKT phosphorylation at Ser473 (Fig. S1*A*). Moreover, treatment of SH-SY5Y cells with a dual mTORC1/mTORC2 inhibitor, KU0063794 (Fig. 2*A*, *lane* 3), or rapamycin and KU0063794 together (Fig. 2*A*, lane 4), produced a similar reduction in phosphorylation of Unkempt as treatment with rapamycin alone (Fig. 2*A*, *lane* 5), further evidence that it is the activity of mTORC1 but not mTORC2 that regulates Unkempt phosphorylation. Furthermore, treatment of SH-SY5Y cells with a range of concentrations of the highly selective mTORC1 inhibitor DL001 (27), which does not inhibit mTORC2-dependent AKT phosphorylation, decreased the mobility of Unkempt and reduced the recognition by an Unkempt phospho-specific antibody (Fig. S1*B*). We also asked whether Unkempt phosphorylation might be regulated at the level of S6K; however, treatment of SH-SY5Y cells with the S6K inhibitor PF4708671 had no noticeable effect (Fig. 2*B*). Together, these data confirm that Unkempt phosphorylation is mTORC1 dependent.

### Unkempt physically interacts with mTORC1

Unkempt has been previously shown to physically interact with Raptor (24, 28). To further test whether Unkempt physically interacts with mTORC1, we used HeLa S3 cells with a doxycycline (DOX)-inducible FLAG-hemagglutinin (HA)-Unkempt to immunoprecipitate Unkempt (23). Rapamycin treatment showed that phosphorylation of FLAG-HA-Unkempt is mTORC1 dependent (Fig. 2*C*). Endogenous Raptor and mTOR coimmunoprecipitated with FLAG-HA-Unkempt (Fig. 2*C*). Unkempt therefore physically interacts with mTORC1.

We also noticed that the coimmunoprecipitation of mTOR in this experiment was sensitive to the type of detergent, whereas pulldown of Raptor was not (Fig. 2*C*). This is in line with a known property of the zwitterionic detergent CHAPS to preserve and the nonionic detergent Triton X-100 to disrupt the interaction between Raptor and mTOR (29), consistent with Unkempt interacting with mTOR indirectly *via* Raptor. These coimmunoprecipitation data further validate the previously observed physical interaction of Unkempt with Raptor (24, 28) and show that Unkempt physically interacts with a functional mTORC1.

### mTORC1 phosphorylates Unkempt at multiple sites in its IDR

**Figure 3:**
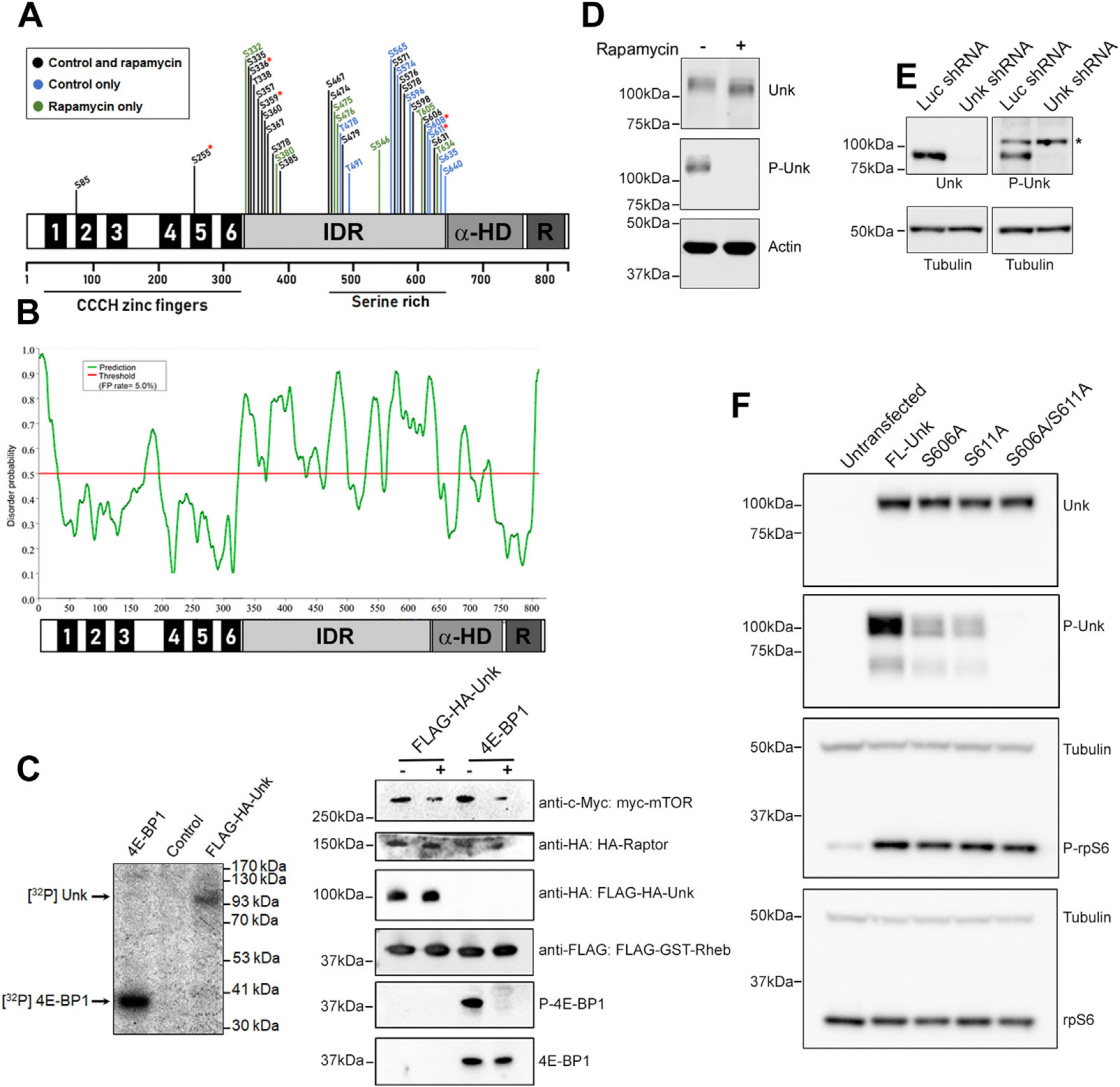
**Unkempt is a direct substrate of mTORC1 and is phosphorylated in the IDR by mTORC1.***A*, a schematic of the phosphorylated residues identified in Unkempt by LC–MS/MS. Phosphorylated residues identified in both control and rapamycin-treated cells are shown in *black*; phosphorylated residues identified only in control cells are shown in *blue*; phosphorylated residues identified only in rapamycin-treated cells are shown in *green*. Phosphorylated residues identified only in the control *in vitro* kinase assay condition and not in the rapamycin/FKBP12 condition are highlighted with a *red asterisk*. See Figs. S2 and S3 for details. *B*, prediction disorder probability plot of the primary sequence of mouse Unkempt. The serine-rich region (amino acids 467–640) is highly disordered. False-positive rate of 5%. *C*, *left hand panel* shows that FLAG-HA-Unkempt purified from HeLa S3 cells is phosphorylated *in vitro* by reconstituted mTORC1/Rheb-GTP. Recombinant 4E-BP1 was used as a positive control. Mock purified extract from uninduced HeLa S3 cells was used as a negative control. *Right hand panel* shows a representative immunoblot of the proteins used for the *in vitro* kinase assays. *D*, a phospho-specific antibody (P-Unk) raised against phospho-S606/phospho-S611 in Unkempt does not recognize Unkempt from rapamycin-treated SH-SY5Y cells. SH-SY5Y cells were grown in DMEM + 10% fetal bovine serum (FBS), then serum starved for 16 h in DMEM alone, then incubated for 1 h in DMEM ± 1 μM rapamycin. *E*, the P-Unk antibody recognizes phosphorylated Unkempt in SH-SY5Y cells transfected with a control shRNA targeting firefly luciferase but not in SH-SY5Y cells transfected with an shRNA targeting Unkempt. *F*, recognition of Unkempt phosphorylation by the P-Unk antibody is reduced or completely lost in HEK293 cells expressing S606A, S611A, or S606/S611A Unkempt mutants. Cells were cotransfected with a plasmid expressing constitutively active Rheb (Rheb^CA^) to activate mTORC1. DMEM, Dulbecco’s modified Eagle’s medium; α-HD, alpha-helical domain; HEK293, human embryonic kidney 293 cell line; IDR, intrinsically disordered region; mTORC1, mechanistic target of rapamycin complex 1; P-Unk, Unkempt phospho-specific antibody; R, RING domain.

Using mass spectrometry (MS) of FLAG-HA-Unkempt immunoprecipitated from cells treated or not with rapamycin, we identified 31 phosphorylated serines and five phosphorylated threonines grouped in three clusters within a predicted IDR of Unkempt (Figs. 3, *A* and *B*, S2 and S3). About 24% of the C-terminal portion of the IDR between amino acids 467 and 640 consists of serine residues (41 residues) and harbors the majority of phosphorylation sites (phosphosites), including all the phosphosites that were never detected in rapamycin-treated cells (Fig. 3*A*, *blue residues*, Figs. S2 and S3). To determine if any of the phosphosites might be direct substrates of mTORC1, we used an *in vitro* kinase assay with purified reconstituted mTORC1 and GTP-bound Rheb. We found that purified Unkempt was indeed directly phosphorylated by mTORC1/Rheb-GTP (Figs. 3*C* and S3*B*). MS identified five serine residues in Unkempt (S255, S336, S359, S608, and S611) that were *in vitro* phosphorylated in the control condition but not in the rapamycin/FKBP12-treated condition (addition of the rapamycin-binding partner FKBP12 is necessary for mTORC1 inhibition *in vitro* (30); Fig. 3*A*, *red asterisks*; Figs. S2 and S3). Four of these serines have a proline or leucine at the +1 position and so adhere to the predicted mTORC1 phosphorylation motif consensus sequence (6, 8), and two (S608 and S611) are also mTORC1 dependent in rapamycin-treated cells (Figs. 3*A*, S2 and S3).

Unkempt phosphorylation at S611 was reproducibly rapamycin dependent in cells (in three biological replicates) and in the *in vitro* kinase assay (Fig. S3). S611 also has a leucine residue at the +1 position and hydrophobic residues at −1 (glycine) and −4 (alanine) and so adheres to the mTORC1 phosphorylation motif consensus sequence (6, 8) (Fig. S2). S611 was therefore the best candidate for an mTORC1-dependent phosphosite in Unkempt. We generated an Unkempt phospho-specific antibody (P-Unk) against a peptide containing phospho-S611 and nearby phospho-S606, which was also reproducibly identified in cells by MS (Fig. S3) and had to be included in the peptide immunogen because of its proximity to S611. This antibody recognized Unkempt in serum-stimulated SH-SY5Y cells but not in cells that were treated with rapamycin (Figs, 3*D* and S1*A*). The P-Unk antibody did not recognize phosphorylated Unkempt in SH-SY5Y cells in which Unkempt has been knocked down using an shRNA (Fig. 3E). Moreover, mutation of S606 or S611, separately or together, strongly reduced or completely prevented the P-Unk antibody from recognizing mTOR-dependent Unkempt phosphorylation (Fig. 3F). Taken together, these data provide evidence that Unkempt is an mTORC1 substrate that is phosphorylated at multiple residues, located mostly in the serine-rich region of the IDR.

**Figure 4:**
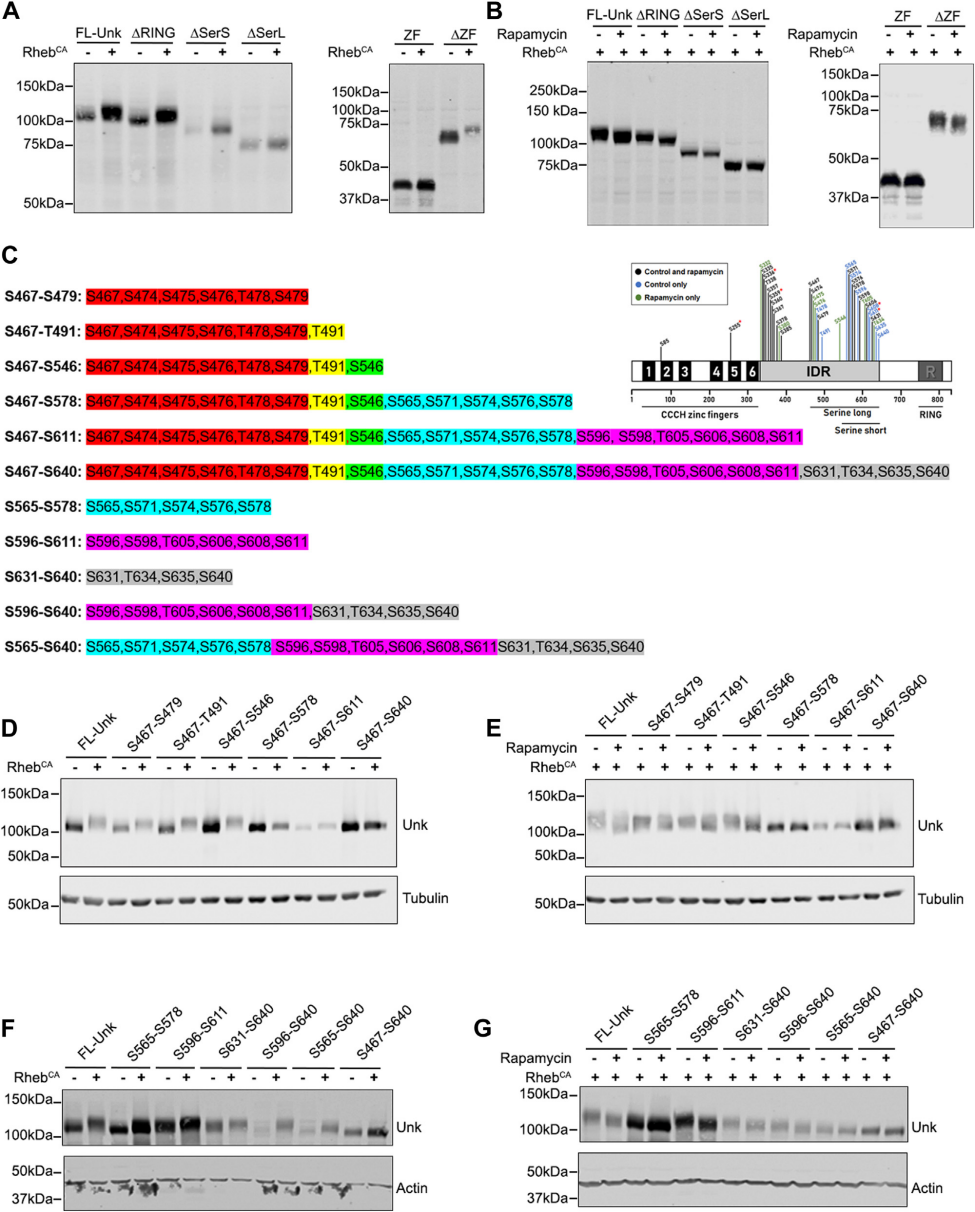
**mTORC1-dependent phosphorylation of Unkempt occurs within the serine-rich region located in the IDR.***A* and *B*, full-length V5-Unkempt (FL-Unk) or deletion mutants lacking the zinc finger domain (ΔZF) or the RING domain (ΔRING) are phosphorylated in HEK293 cells expressing constitutively active Rheb (Rheb^CA^) (*A*) and dephosphorylated in these cells treated with rapamycin (*B*). Phosphorylation is absent with ZF domain alone (ZF) and completely or partially absent with deletion mutants lacking whole serine-rich region (ΔSerL) or part of the serine-rich region (ΔSerS) (*A* and *B*). *C*, the serine and threonine residues that were mutated to alanine in the Unkempt mutants used in *D*–*G*. *D* and *E*, mutations to alanines of the increasingly larger number of phosphosites within IDR leads to a progressively lower capacity of the constitutively active Rheb (Rheb^CA^) to phosphorylate Unkempt. Full-length V5-Unkempt (FL-Unk) or FL-Unk containing alanine mutations in phosphorylated serine and threonine residues between S467–S546 expressed in HEK293 cells are phosphorylated by Rheb^CA^ (*D*) and dephosphorylated by rapamycin (*E*). Additional mutation of phosphorylated residues from S467–S578, S467–S611, and S467–S640 partially or completely prevent phosphorylation by constitutively active Rheb (*D*) and dephosphorylation by rapamycin (*E*). *F* and *G*, alanine mutants of Unkempt in phosphorylated serine and threonine residues between S565 and S640 are phosphorylated by constitutively active Rheb (Rheb^CA^) (*F*) and dephosphorylated by rapamycin (*G*). HEK293 cells were grown in DMEM + 10% fetal bovine serum (FBS), then serum starved for 16 h in DMEM alone, and then incubated for 1 h in DMEM ± 1 μM rapamycin. DMEM, Dulbecco’s modified Eagle’s medium; α-HD, alpha-helical domain; HEK293, human embryonic kidney 293 cell line; IDR, intrinsically disordered region; mTORC1, mechanistic target of rapamycin complex 1.

To independently define the region of Unkempt that is targeted by mTORC1-dependent phosphorylation, we performed deletion analysis of Unkempt transiently expressed in human embryonic kidney 293 cells. Coexpression of constitutively active Rheb, which directly hyperactivates mTORC1 (31), caused phosphorylation of full-length Unkempt in the absence but not in the presence of rapamycin (Fig. 4, *A* and *B*). Unkempt deletion mutants lacking either the ZF domain (ΔZF) or the RING domain (ΔRING) retained mTORC1-dependent phosphorylation potential, whereas a mutant containing just the ZF domain alone (ZF) was not phosphorylated by mTORC1 hyperactivation (Fig. 4, *A* and *B*). Notably, removal of the whole serine-rich region (ΔSerL) or of a shorter region between amino acids 539 and 640 (ΔSerS) almost completely prevented phosphorylation (Fig. 4, *A* and *B*). This deletion analysis confirms that the serine-rich region in Unkempt contains the majority of the mTORC1-dependent phosphosites.

MS analysis showed that specific serine and threonine residues in the IDR of Unkempt are targets of mTORC1-dependent phosphorylation (Fig. 3*A*). To test these data further, we mutated pairs of mTORC1-dependent serine residues in the IDR (S565/S574, S608/S611, and S635/S640) to alanine. However, mutation of these serine pairs did not prevent Unkempt phosphorylation (Fig. S3*C*). Therefore, we took a systematic approach and generated a series of mutants in which clusters of MS-identified phosphorylated serine and threonine residues in the IDR were mutated to alanine (Fig. 4*C*). Mutation of phosphosites from S467 to S546 to alanine had no obvious effect on mTORC1-dependent phosphorylation of Unkempt (Fig. 4, *D* and *E*). However, additional mutation of phosphosites between S565–S578, S596–S611, and S631–S640 to alanine strongly reduced mTORC1-dependent phosphorylation of Unkempt (Fig. 4, *D* and *E*). Based on this, we then tested mutants that only affect phosphosites located exclusively within the serine-rich region of the IDR (Fig. 4*C*). Interestingly, mutations of groups of these phosphosites (S565–S578, S596–S611, or S631–S640), or their combinations (S596–S640 or S565–S640), reduced but did not completely prevent the overall mTORC1-dependent phosphorylation of Unkempt (Fig. 4, *C*, *F* and G). These mutagenesis data validate the MS analyses and point to the serine-rich region of the IDR as the hot spot of mTORC1-dependent phosphorylation of Unkempt.

### Unkempt is phosphorylated by mTORC1 *in vivo*

**Figure 5:**
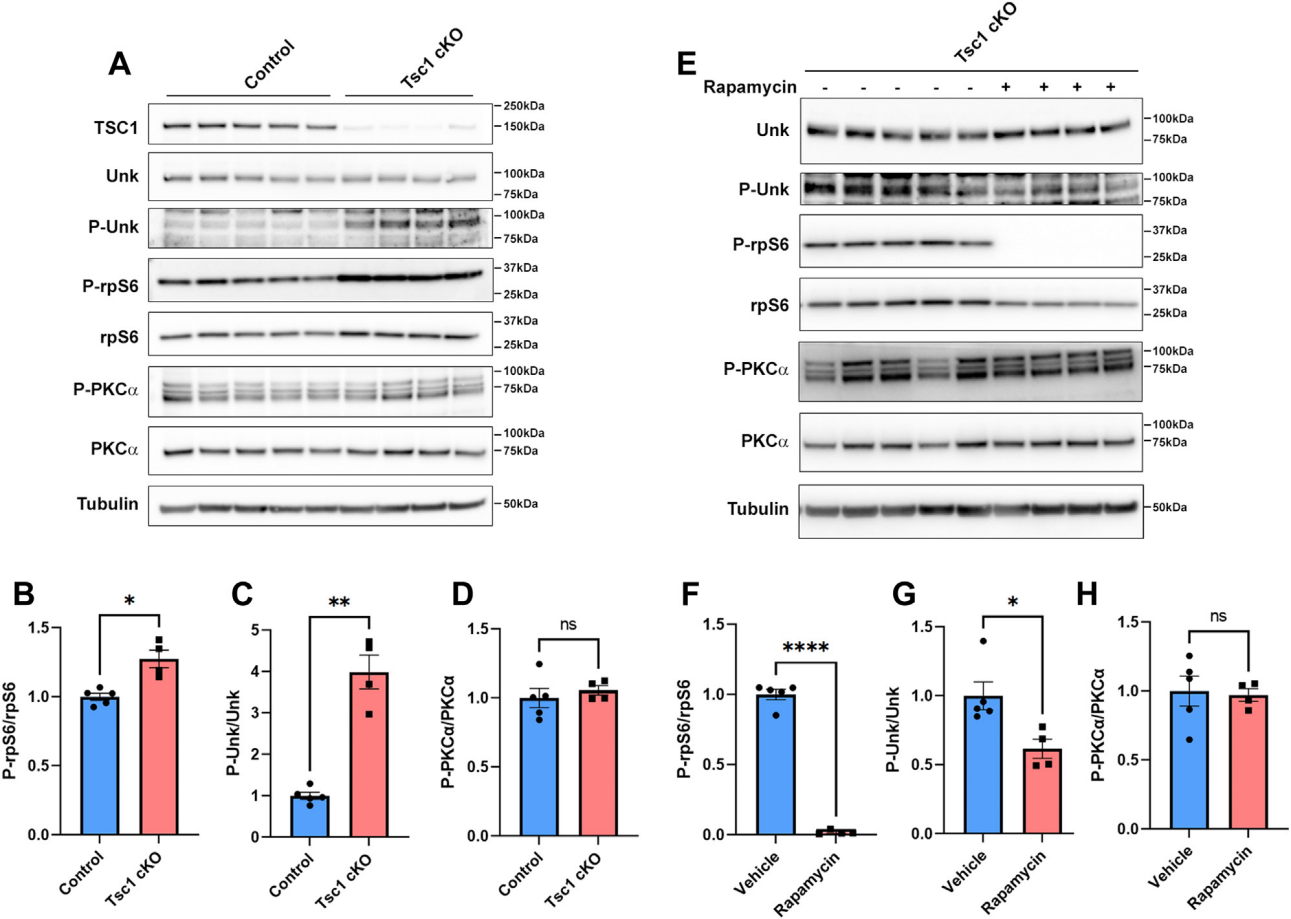
**Unkempt is phosphorylated by mTORC1 during neurodevelopment.***A*, Western blot analysis of E16.5 brain lysate from control (*Nestin-Cre;Tsc1*^*fl/+*^) and Tsc1 cKO (*Nestin-Cre;Tsc1*^*fl/fl*^) mice. *B*–*D*, quantification of phospho-rpS6 (*B*), phospho-Unkempt (*C*), and phospho-PKCα (*D*) expression. Control, n = 5; Tsc1 cKO, n = 4. *E*, Western blot analysis of E16.5 brain lysate from Tsc1 cKO (*Nestin-Cre;Tsc1*^*fl/fl*^) mice treated with vehicle or rapamycin for 24 h. *F*–*H*, quantification of phospho-rpS6 (*F*), phospho-Unkempt (*G*), and phospho-PKCα (*H*) expression. Vehicle, n = 5; rapamycin treated, n = 4. Data are represented as mean ± SEM. Student’s *t* test, ns, ∗*p* < 0.05, ∗∗*p* < 0.01, and ∗∗∗∗*p* < 0.0001. cKO, conditional KO; mTORC1, mechanistic target of rapamycin complex 1; ns, not significant.

In mice, Unkempt protein is expressed in the developing nervous system from at least as early as E10 (23). To analyze the phosphorylation status of Unkempt by mTORC1 *in vivo*, we utilized *Nestin-Cre;Tsc1*^*fl/fl*^ mice, in which *Nestin* promoter–driven Cre recombinase is expressed in neural progenitors beginning at E10.5 causing loss of TSC1 expression throughout the developing brain (32). Consequently, *Nestin-Cre;Tsc1*^*fl/fl*^ mice exhibit activated mTORC1 signaling in the brain, macrocephaly, and die at P0, likely because of malnutrition, hypoglycemia, and hypothermia (32). The activated mTORC1 signaling and perinatal death of these mice can be suppressed by a single dose of rapamycin between E15 and E17 (32). We confirmed hyperactivation of mTORC1 *via* increased phosphorylation of rpS6 in the brain of E16.5 *Nestin-Cre;Tsc1*^*fl/fl*^ mice embryos (Fig. 5, *A* and *B*). Although we did not detect a change in the electrophoretic mobility of Unkempt protein, using the phospho-specific antibody, we observed a dramatic increase in Unkempt phosphorylation in brain tissue from *Nestin-Cre;Tsc1*^*fl/fl*^ embryos (Fig. 5, *A* and *C*).

To test whether inhibition of mTORC1 affects Unkempt phosphorylation during nervous system development, mice pregnant with *Nestin-Cre;Tsc1*^*fl/fl*^ embryos were injected with rapamycin or vehicle at E15.5. Twenty-four hours after rapamycin injection, rpS6 phosphorylation was dramatically reduced in the embryonic brain (Fig. 5, *E* and *F*). Rapamycin treatment also significantly decreased phosphorylation of Unkempt (Fig. 5, *E* and *G*). To determine whether prolonged rapamycin treatment affected mTORC2 signaling (13), we analyzed PKCα phosphorylation (33). PKCα phosphorylation was unaffected in the brain of *Nestin-Cre;Tsc1*^*fl/fl*^ embryos compared with controls (Fig. 5, *A* and *D*) and did not change with 24-h rapamycin treatment (Fig. 5, *E* and *H*), indicating that mTORC2 signaling was unaffected. These data show that Unkempt phosphorylation is mTORC1 dependent in the developing brain.

### Phosphorylation of Unkempt affects its capacity to remodel cellular morphology

**Figure 6:**
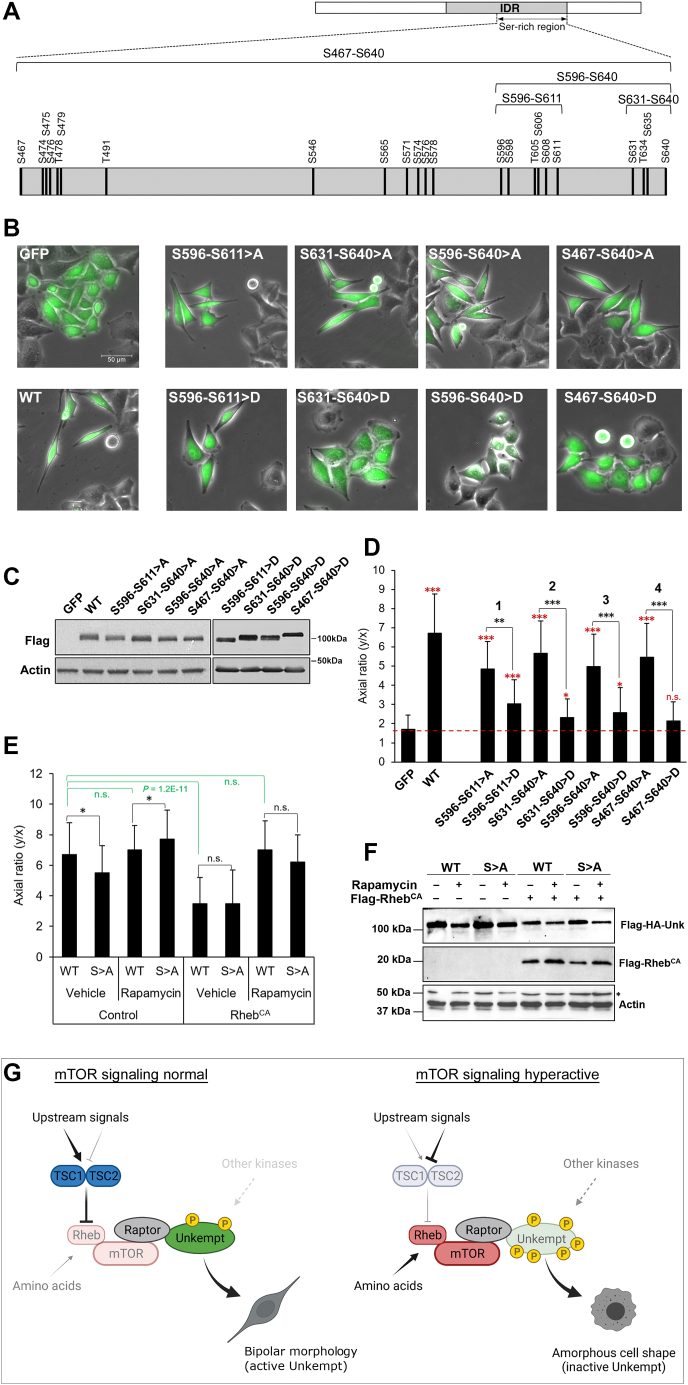
**Phosphosite mutationsin the serine-rich region compromise the morphogenetic activity of Unkempt.***A*, map of Unkempt protein (*top*) with the locations of all detected phosphosites within the serine-rich region of the IDR shown magnified on the *bottom*, drawn to scale. *B*, GFP-only–inducible or GFP and WT or phosphosite alanine (>A) or aspartate (>D) mutant Unkempt-inducible HeLa cells at 48 h of treatment with doxycycline. The scale bar represents 50 μm. *C*, detection by immunoblotting of WT or the indicated mutant Unkempt proteins in total cell lysates of inducible HeLa cells at 48 h of treatment with doxycycline. *D*, the morphologies of the HeLa cell cultures indicated in (*B*) were quantified by calculating their axial ratios (*y*/*x*), as described previously (see Experimental procedures section (23)). The results are compared with the GFP control (*red asterisks*) morphology, or mutant Unkempt-expressing cells are compared for each phosphosite region indicated in (*A*) (*black asterisks*). *Red dashed line* denotes the average axial ratio of the GFP control cells. Error bars indicate SD (n = 50). (∗) 4.3E-5 < *p* < 5.2E-5; (∗∗) *p* = 3.9E-6; (∗∗∗) *p* < 1.3E-10 (pertains to *black* and *red asterisks*), Student’s *t* test. ns. *E*, mTORC1-mediated phosphorylation modulates Unkempt-induced cell morphogenesis. GFP and WT or S467–S640 > A mutant (S > A) Unkempt-inducible HeLa cells stably expressing Rheb^CA^, or not (control), were incubated for 48 h with doxycycline and either without (vehicle) or with 100 nM rapamycin, as indicated. Morphologies of 50 GFP-expressing cells were quantified for each condition as in (*D*). Error bars indicate SD (n = 50). (∗) 1.1E-3 < *p* < 9.0E-3, Student’s *t* test. *F*, detection by immunoblotting of the indicated proteins in total cell lysates for each of the conditions analyzed in (*E*). (∗) Nonspecific band. *G*, a model for the regulation of cellular morphogenesis by Unkempt acting downstream of mTORC1. Created with BioRender.com. IDR, intrinsically disordered region; mTORC1, mechanistic target of rapamycin complex 1; ns, not statistically significant.

In addition to being required for the early morphology of neurons, Unkempt also possesses a remarkable capacity to establish a similar and early neuronal-like bipolar morphology in cells of non-neuronal origin, indicating that Unkempt regulates a specific program of cell morphogenesis (22, 23). To examine whether phosphorylation affects the morphogenetic activity of Unkempt, we inducibly expressed in HeLa cells GFP either alone or together with wildtype or phosphosite-mutant Unkempt and monitored the effects on cell shape. With clusters of phosphosites turned to either alanines or aspartates to prevent or mimic phosphorylation, respectively, we focused on the serine-rich region of IDR, in particular its C-terminal region (S596–S640), as the hot spot for mTORC1-dependent phosphorylation of Unkempt (Figs. 4, *C*–*G* and 6*A*). Strikingly, whereas alanine mutations largely preserved the morphogenetic activity of Unkempt, aspartate mutations severely compromised or abolished cell polarization by Unkempt (Figs. 6, *B*–*D* and S4). All mutants showed similar gross RNA binding to wildtype Unkempt (Fig. S5), suggesting that the morphogenetic effects of these mutations are unlikely to be due to differences in RNA binding, a key requirement for Unkempt-induced cell morphogenesis (22, 23). Moreover, we found that mTORC1 hyperactivation through expression of constitutively active Rheb significantly attenuated the morphogenetic activity of Unkempt, an effect that could be rescued by treatment with rapamycin (Fig. 6, *E* and *F*). Curiously, the S467–S640>A mutant still responded to changes in mTORC1 activity, similarly to the wildtype Unkempt (Fig. 6*E*). This result suggests that none of the 23 serine/threonine residues, which we turned to alanines in this mutant (Fig. 4*C*), are strictly required to confer sensitivity of Unkempt to mTORC1. Instead, we suspect that regulation of morphology by mTORC1 could occur through phosphorylation of other residues in Unkempt (there are an additional 30 serine/threonine residues present just in the disordered serine-rich region alone) and/or through phosphorylation of residues in its interacting proteins. Together, these data point to a critical role of mTORC1-dependent phosphorylation in regulating the activity of Unkempt in cellular morphogenesis (Fig. 6*G*). These studies pave the way for investigation of the mechanistic principles by which Unkempt and its phosphorylation by mTORC1, and potentially other kinases, regulate cellular morphogenesis.

## Discussion

Here, we have shown that phosphorylation of the highly conserved ZF/RING domain protein Unkempt is regulated by mTORC1 *in vitro* and in the embryonic brain. Unkempt is a direct substrate of and physically interacts with mTORC1 *via* binding to Raptor. mTORC1-dependent phosphorylation of Unkempt was shown by MS to occur on multiple serine and threonine residues concentrated in the IDR. Using extensive mutagenesis, we showed that mTORC1-dependent phosphorylation of Unkempt requires residues in the serine-rich region of the IDR. Importantly, we showed that phosphomimetic mutations in this serine-rich region abrogate the ability of Unkempt to induce a bipolar morphology. Taken together, our findings show that Unkempt is a novel substrate of mTORC1 and that mTORC1-dependent phosphorylation acutely regulates Unkempt’s ability to remodel cellular morphogenesis (Fig. 6*G*).

mTORC1 regulates the phosphorylation of hundreds of different proteins (6, 7, 8, 10) with roles in growth control, autophagy, nucleotide and lipid metabolism, but the control of cellular morphogenesis by mTORC1 is less well characterized. The microtubule-associated proteins CLIP-170 and Tau are known to be direct substrates of mTORC1 but do not have the Unkempt’s profound ability to reorganize cell morphology (34, 35). We have shown that Unkempt phosphorylation is exquisitely sensitive to mTORC1 activity and is modulated by a range of inputs including glucose, amino acids, and insulin signaling. In the developing brain, mTORC1-dependent Unkempt phosphorylation, detected by mobility shift, appears much less than *in vitro*, although phosphorylation detected using a phospho-specific antibody is still highly sensitive to mTORC1 activity *in vivo*. During nutrient-deprived conditions, the nervous system is preferentially spared at the expense of other organs to maintain brain growth (36, 37). The range of potential mTORC1 activity in the developing nervous system may therefore be less than in cultured cells, potentially explaining the differences in the level of mTORC1-dependent Unkempt phosphorylation. Moreover, additional kinases and phosphatases may regulate Unkempt phosphorylation *in vivo*.

Raptor physically interacts with Unkempt, enabling mTOR to phosphorylate multiple residues within the serine-rich region of the Unkempt IDR. Multiple independent studies have also demonstrated a physical interaction between Unkempt and Raptor (24, 28, 38, 39, 40). Physical interaction between endogenous Unkempt and Raptor has not yet been shown, possibly because the interaction of endogenous Unkempt with mTORC1 is transient and difficult to detect. Unkempt phosphorylation is complex and includes mTORC1-dependent and -independent serine and threonine residues, suggesting the involvement of additional kinases. The number and proximity of phosphosites in the IDR also implies potential flexibility in the regulation of Unkempt phosphorylation, a characteristic of IDRs (41). *In vivo* phosphorylation of Unkempt may depend on the context, both spatially and temporally. Thus, through phosphorylation, the IDR of Unkempt might serve as a rheostat that integrates inputs from multiple signaling pathways to fine-tune the activity of Unkempt (Fig. 6*G*). This might not be limited to the control of the cellular morphogenesis but could extend to other known roles of Unkempt in neuronal differentiation and cell proliferation (18, 21, 24, 28, 42, 43).

Our results suggest that mTORC1-mediated phosphorylation controls Unkempt-induced morphogenesis in a manner similar to the effect of aspartate mutations of Unkempt (Fig. 6, *A*–*F*). However, despite this and our evidence that Unkempt is directly phosphorylated by mTORC1 (Fig. 3), we cannot conclude that phosphorylation of Unkempt by mTORC1 definitely controls cellular morphogenesis. Although our data suggest that this is a distinct possibility, mTORC1 could, in principle, control Unkempt-dependent morphogenesis through phosphorylation of another protein that might work together with Unkempt. In the latter scenario, the observed direct phosphorylation of Unkempt by mTORC1 could serve another, unknown Unkempt-regulated process occurring concurrently with cell morphogenesis.

It remains unclear how phosphorylation might regulate the activity of Unkempt at the molecular level. Thus far, the most extensively documented molecular property of Unkempt is its capacity to bind mRNA, which occurs in an RNA sequence–specific manner and is mediated by a tandem array of six highly conserved CCCH-type ZFs located at the N terminus (22, 23). Through RNA binding, Unkempt is thought to primarily regulate the translation of several hundred of its target mRNAs involved in processes including control of protein synthesis and cell morphogenesis, although the mechanistic basis for translational control and potential other modes of post-transcriptional processing by Unkempt remains unknown. Given the physical separation of the ZF domain from the heavily phosphorylated serine-rich region in the IDR, but also because the ZF domain alone suffices for high-affinity RNA binding, it seems unlikely that phosphorylation in the serine-rich region would affect RNA binding by Unkempt (22, 23). Indeed, our mutagenesis analysis coupled with crosslinking and immunoprecipitation (CLIP) data suggests that phosphorylation exerts no overt effect on Unkempt–RNA interactions. Instead, we propose that phosphorylation by mTORC1 acts to modulate protein–protein interactions (PPIs) between the IDR and partner proteins, in keeping with the known propensity of IDRs to act as protein rheostats by forming transient PPIs regulated by phosphorylation (41). Phosphorylation-sensitive PPIs might entail interactions of Unkempt with a large number of cytosolic RNA granule components and other effectors of post-transcriptional processing identified *via* unbiased screens (44, 45, 46). Such a mechanism could provide a novel route for the mTOR pathway to control morphogenesis and other cellular processes, beyond those regulated by 4E-BP/eIF4E and S6K (47).

## Experimental procedures

### Animal models

Mouse studies were carried out in accordance with UK Home Office regulations and the UK Animals (Scientific Procedures) Act of 1986 (ASPA) under a UK Home Office license (PPL 70/8719) and approved by the King’s College London Ethical Review Committee. *Nestin-Cre* mice (obtained from Jackson; stock number 003771) and *Tsc1*^*fl/fl*^ mice (obtained from Jackson; stock number 005680) were maintained in a C57BL/6 background. Mice were housed under a 12 h light/dark cycle with ad libitum access to food and water. *Tsc1*^*fl/fl*^ mice were genotyped using primers 5′-GTC ACG ACC GTA GGA GAA GC-3′ and 5′-GAA TCA ACC CCA CAG AGC AT-3′. *Nestin-Cre* mice were genotyped using primers 5′-TTGCTAAAGCGCTACATAGGA-3′ and 5′-GCCTTATTGTGGAAGGACTG-3′.

Pregnant dams were injected intraperitoneally with 1 mg/kg rapamycin (Fluorochem Ltd) at E15.5, embryos removed at E16.5, and embryonic brains dissected and immediately frozen on dry ice.

### Cell culture, immunoprecipitation, and Western blot analysis

SH-SY5Y, Neuro-2a, and human embryonic kidney 293 cells were maintained in Dulbecco’s modified Eagle’s medium high glucose (Sigma–Aldrich) supplemented with 10% (v/v) fetal bovine serum (Sigma–Aldrich) and 1% (w/v) penicillin/streptomycin (Sigma–Aldrich). Details of culture conditions, drug treatments, Western blot, and antibodies are described in the supporting information.

### Expression vectors, cloning, and transfections

Details of DNA constructs and transfections are described in the supporting information.

### Coimmunoprecipitation experiments and LC–MS/MS analysis

To induce overexpression of and immunoprecipitate FLAG-HA-Unkempt, previously described HeLa S3 cells stably expressing DOX-inducible FLAG-HA-Unkempt (23) were used. Details of coimmunoprecipitation experiments are described in the supporting information. LC–MS/MS analysis was performed by the Cambridge Centre for Proteomics, University of Cambridge; details are described in the supporting information.

### *In vitro* mTORC1-Rheb kinase assays

Radiolabeled *in vitro* mTORC1 kinase assays with Rheb-GTP were performed as described previously (30). *In vitro* mTORC1 kinase assays for LC–MS/MS are described in the supporting information.

### Immunofluorescence and immunohistochemistry

Details of immunofluorescence and immunohistochemistry are described in the supporting information.

### Unkempt phospho-specific antibody generation

Three phosphopeptides were generated corresponding to residues 601 to 610 (containing phosphoserine 606), 607 to 615 (containing phosphoserine 611), and 601 to 615 (phosphoserines 606 and 611) in Unkempt. Rabbits were immunized with all three phosphopeptides, and phospho-specific antibodies were affinity purified using the phosphopeptide corresponding to residues 601 to 615 (containing phosphoserines 606 and 611). Antibody generation was performed by Covalab.

### Morphogenesis assays

Cellular morphologies were analyzed as described previously (27). Briefly, the morphology of each cell was evaluated by an axial ratio (*y*/*x*), where the length of the absolute longest cellular axis (*y*) was divided by the length of the longest axis perpendicular to the *y*-axis (*x*). Lengths of *x*- and *y*-axes of GFP-positive–inducible HeLa cells were measured at 48 h of treatment with DOX. At least 50 GFP-positive cells were quantified for each inducible cell line.

### UV-CLIP assays

CLIP experiments were carried out essentially as described (48). Further details are described in the supporting information.

### Statistical analyses

Data were analyzed using GraphPad Prism, version 7 (GraphPad Software, Inc). Data distributions were assessed for normality and then analyzed using a Student’s *t* test. *p* Values <0.05 were considered significant.

## Materials and data availability

All methods and data are described in the article or supporting information.

## Supporting information

This article contains supporting information (23, 29, 30, 31, 48, 49, 50, 51)

## Conflict of interest

The authors declare that they have no conflicts of interest with the contents of this article.

## Supplementary Data

Supplemental Material

This work was funded by a Health Schools PhD studentship from 10.13039/100009360King’s College London (to E. V.). The 10.13039/100010269Wellcome Trust (grant no.: 200668/Z/16/Z) and the 10.13039/100000005USA Department of Defense (grant no.: W81XWH-17-1-0082) (to J. M. B.). 10.13039/100012068Health and Care Research Wales (the Wales Cancer Research Centre) and the 10.13039/501100000311Tuberous Sclerosis Association supported A. R. T. S. K. U. and S. R. M. received funding from the 10.13039/100010438Francis Crick Institute, which receives its core funding from 10.13039/501100000289Cancer Research UK (grant no.: FC001201), the 10.13039/501100000265UK Medical Research Council (grant no.: FC001201), and the 10.13039/100010269Wellcome Trust (grant no.: FC001201). The 10.13039/100000057National Institute of General Medical Sciences (grant no.: GM144693) and the 10.13039/100014802University of California Research Initiatives grant (grant no.: CRN-20-637684) provided support to J. M.
