# Supplementary material for: Phosphorylation of the novel mTOR substrate Unkempt regulates cellular morphogenesis
Source: J Biol Chem. 2022 Dec 9;299(1):102788. doi: 10.1016/j.jbc.2022.102788 (PMC9852543; doi:10.1016/j.jbc.2022.102788)
Supplement: Supplemental Material [file mmc1.docx]

**Supplemental Figures**

**
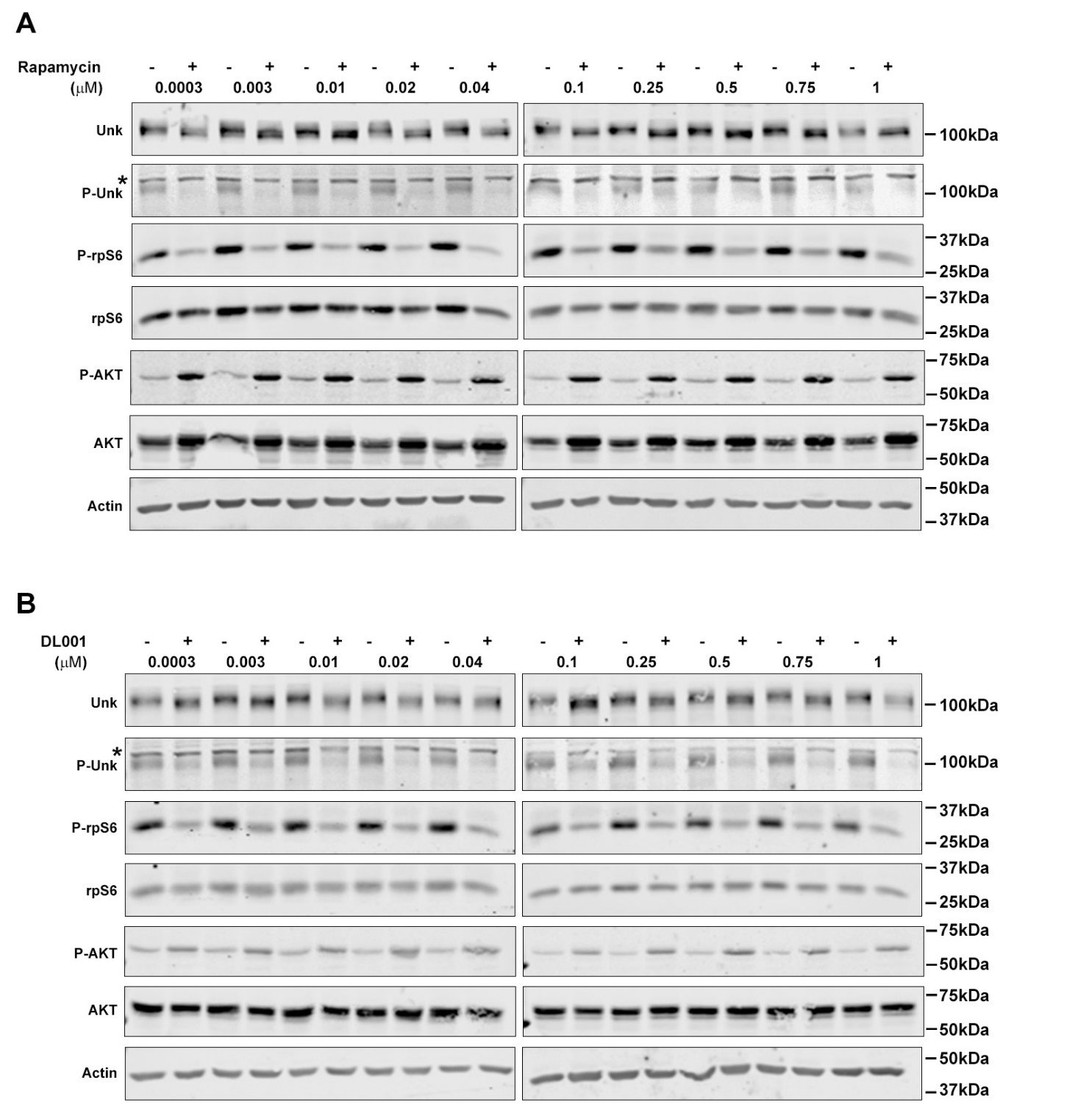
**

*Figure S1. Unkempt phosphorylation is highly sensitive to mTORC1 inhibition.* (A) The electrophoretic mobility of Unkempt in SH-SY5Y cells is decreased by a wide range of rapamycin concentrations. P-Unk is probed using an Unkempt phospho-S606/phospho-S611 specific antibody, see text for details. *indicates a non-specific band. (B) Unkempt phosphorylation in SH-SY5Y cells is inhibited by a wide of concentration of the highly selective mTORC1 inhibitor DL001. *indicates a non-specific band. SH-SY5Y cells were grown in DMEM + 10% foetal bovine serum (FBS), then serum-starved for 16 hours in DMEM alone, then incubated for 1 hour in DMEM ± rapamycin or DL001 at the concentrations shown.

Mm MSKGPGPGGSAASSAPPAATAQVLQAQPEKPQHYTYLKEFRTEQCPLFVQHKCTQHRPYT 60

Hs MSKGPGPGGSAASSAPPAATAQVLQAQPEKPQHYTYLKEFRTEQCPLFVQHKCTQHRPYT 60

Dm -------------MLANETNKLLLSSQQEKPNHYTYLKEFRVEQCQSFLQHKCNQHRPFV 47

. :. :*.:* ***:*********.*** *:****.****:.

Mm CFHWHFVNQRRRRSIRRRDGTFNYSPDVYCTKYDEATGLCPEGDECPFLHRTTGDTERRY 120

Hs CFHWHFVNQRRRRSIRRRDGTFNYSPDVYCTKYDEATGLCPEGDECPFLHRTTGDTERRY 120

Dm CFNWHFQNQRRRRPVRKRDGTFNYSADNYCTKYDETTGICPEGDECPYLHRTAGDTERRY 107

**:*** ******.:*:********.* *******:**:********:****:*******

Mm HLRYYKTGICIHETDSKGNCTKNGLHCAFAHGPHDLRSPVYDIRELQAMEALQNGQTTVE 180

Hs HLRYYKTGICIHETDSKGNCTKNGLHCAFAHGPHDLRSPVYDIRELQAMEALQNGQTTVE 180

Dm HLRYYKTCMCVHDTDSRGYCVKNGLHCAFAHGMQDQRPPVYDIKEL---ETLQNAESTLD 164

******* :*:*:***:* *.*********** :* *.*****:** *:***.::*::

Mm GSIEGQSAGAASHAMIEKILSEEPRWQETAYVLGNYKTEPCKKPPRLCRQGYACPYYHNS 240

Hs GSIEGQSAGAASHAMIEKILSEEPRWQETAYVLGNYKTEPCKKPPRLCRQGYACPYYHNS 240

Dm STNALNALDKE-----RNLMNEDPKWQDTNYVLANYKTEPCKRPPRLCRQGYACPQYHNS 219

.: :: . .:::.*:*:**:* ***.********:************ ****

#

Mm KDRRRSPRKHKYRSSPCPNVKHGDEWGDPGKCENGDACQYCHTRTEQQFHPEIYKSTKCN 300

Hs KDRRRSPRKHKYRSSPCPNVKHGDEWGDPGKCENGDACQYCHTRTEQQFHPEIYKSTKCN 300

Dm KDKRRSPRKYKYRSTPCPNVKHGEEWGEPGNCEAGDNCQYCHTRTEQQFHPEIYKSTKCN 279

**:******:****:********:***:**:** ** ***********************

# #

Mm DMQQAGSCPRGPFCAFAHIEPPPLSDDVQPSSAVSSPTQPGPVLYMPSAAGDSVPVSPSS 360

Hs DMQQSGSCPRGPFCAFAHVEQPPLSDDLQPSSAVSSPTQPGPVLYMPSAAGDSVPVSPSS 360

Dm DVQQAGYCPRSVFCAFAHVEPCSMDD---------------------------------- 305

*:**:* ***. ******:* .:.*

Mm PHAPDLSALLCRNSGLGSPSHLCSSPPGPSRKASNLEGLVFPGESSLAPGSYKKAPGFER 420

Hs PHAPDLSALLCRNSSLGSPSNLCGSPPGSIRKPPNLEGIVFPGESGLAPGSYKKAPGFER 420

Dm -----------------------------------------PRENSLS------------ 312

* *..*:

Mm EDQVGAEYLKNFKCQAKLKPHSLEPRSQEQPLLQPKQDVLGILPVGSPLTSSISSSITSS 480

Hs EDQVGAEYLKNFKCQAKLKPHSLEPRSQEQPLLQPKQDMLGILPAGSPLTSSISSSITSS 480

Dm ---------------ASLANTSLLTRSS-APINIPN----------TTLSNSINDFNSGS 346

*.* ** .**. *: *: :.*:.**.. :.*

Mm LAATPPSPAGTNSTPGMNANALPFYPTSDTVESVIESALDDLDLNEFGVAALEKTFDNSA 540

Hs LAATPPSPVGTSSVPGMNANALPFYPTSDTVESVIESALDDLDLNEFGVAALEKTFDNST 540

Dm FAVNIPS------------SSLTYSPTN--------------HANLFNVDAFNYGGSN-K 379

:*.. ** .:*.: **. . * *.* *:: .*

Mm VPHPSSVTIGGSLLQSSAPVNIPGSLGSSASFHSASPSPPVSLSSHFLQQPQGHLSQSEN 600

Hs VPHPGSITIGGSLLQSSAPVNIPGSLGSSASFHSASPSPPVSLSSHFLQQPQGHLSQSEN 600

Dm LSNSLSATQNDSSLFFPSRIISPG-FG-----DGLSISPSVRIS---------ELNTIRD 424

:.:. * * ..* * .: : ** :* .. * **.* :* .*. .:

# #

Mm TFLGTSASHGSLGLNGMNSSIWEHFASGSFSPGTSPAFLSGPGAAELARLRQELDEANGT 660

Hs TFLGTSASHGSLGLNGMNSSIWEHFASGSFSPGTSPAFLSGPGAAELARLRQELDEANST 660

Dm DINSSSVGN-SLFENTLNT------AKNAFSLQS----LQSQNNSDLGRITNELLTKNAQ 473

: .:*..: ** * :*: *..:** : *.. . ::*.*: :** *.

Mm IKQWEESWKQAKQACDAWKKEAEEAGERASAAGAECELAREQRDALELRVKKLQEELERL 720

Hs IKQWEESWKQAKQACDAWKKEAEEAGERASAAGAECELAREQRDALEVQVKKLQEELERL 720

Dm IHKLNG---------------------RFEDMACKLKIAELHRDKAKQEAQEWKERYD-- 510

*:: : * . ..: ::*. :** : ..:: :*. :

Mm HTVPEAQTLPAAPDLEALSLSTLYSIQKQLRVHLEQVDKAVFHMQSVKCLKCQEQTR--A 778

Hs HAGPEPQALPAFSDLEALSLSTLYSLQKQLRAHLEQVDKAVFHMQSVKCLKCQEQKR--A 778

Dm ---LAQIQLNLPAELRDLSIQKLKQLQSKLRTDLEEVDKVLYLENAKKCMKCEENNRTVT 567

* .:*. **:..* .:*.:**..**:***.:: :: **:**:*:.* :

Mm VLPCQHAVLCELCAE-GSECPVCQPSRAHALQS 810

Hs VLPCQHAALCELCAE-GSECPICQPGRAHTLQS 810

Dm LEPCNHLSICNTCAESVTECPYCQVPVITTHT- 599

: **:* :*: *** :*** ** :

*Figure S2. Phosphorylated residues identified in Unkempt.* The primary sequence of *Mus musculus* (Mm) Unkempt aligned with *Homo sapiens* (Hs) and *Drosophila melanogaster* (Dm) Unkempt. Grey highlighted residues were phosphorylated in vehicle control and rapamycin treatment conditions; blue highlighted residues were phosphorylated only in the vehicle control condition; green highlighted residues were phosphorylated only in the rapamycin treatment condition. # indicates residues phosphorylated only in the control condition in the mTORC1 *in vitro* kinase assay. See Supplemental Figure S3 for details.


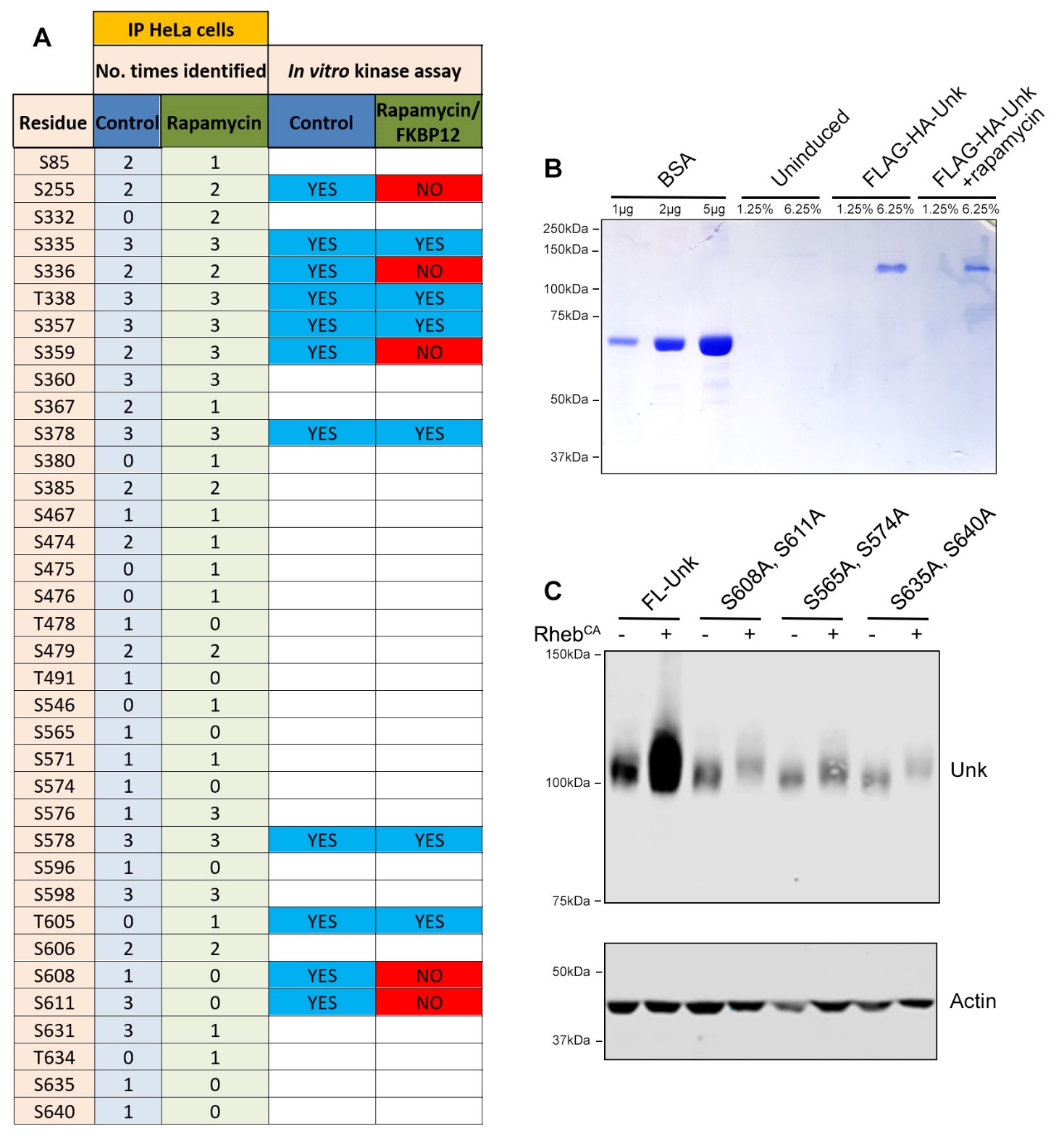


*Figure S3. mTORC1 dependent and independent phosphorylated residues in Unkempt*. (A) Columns 1-3 show a summary of the number of times each phosphorylated residue was identified by LC-MS/MS in FLAG-HA-Unkempt immunoprecipitated from vehicle (DMSO) control and rapamycin treated HeLa S3 cells in three biological replicates. Columns 4 and 5 show phosphorylated residues identified by LC-MS/MS of purified FLAG-HA-Unkempt following an *in vitro* kinase assay with reconstituted mTORC1/Rheb^CA^ in control or rapamycin/FKBP12 conditions. (B) Representative Coomassie stained SDS-PAGE gel of purified FLAG-HA-Unkempt used for the in vitro kinase assays. Indicated are the percentages of the total elution volume loaded on the gel. (C) Full length wildtype V5-Unkempt (FL-Unk) or FL-Unk containing the indicated pairs of serine residues mutated to alanines were expressed in HEK293 cells together with constitutively active Rheb (Rheb^CA^).

**
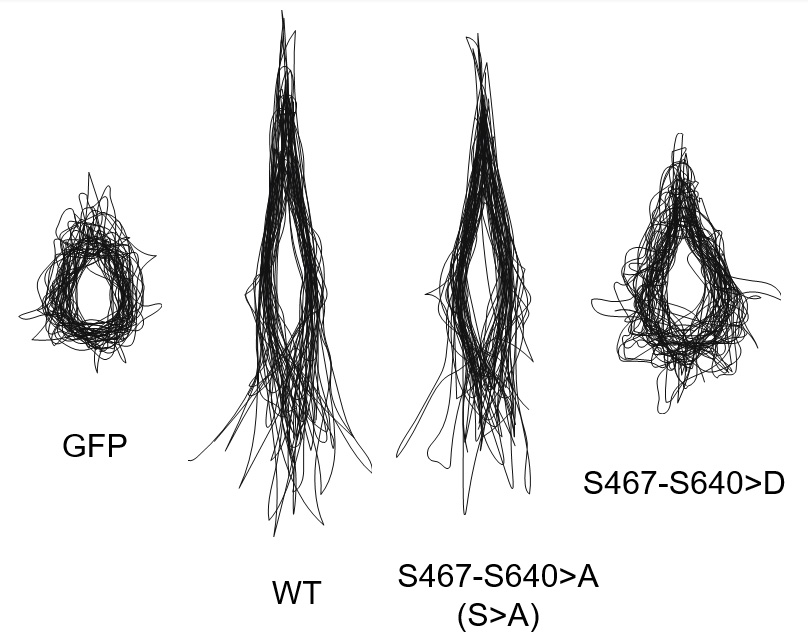
**

*Figure S4.* Overlaid outlines of GFP-only inducible (GFP) or GFP and either wild-type (WT), S467-S640>A, or S467-S640>D Unkempt-inducible HeLa cells at 48 hours of treatment with doxycycline. Outlines of 40 GFP-positive cells were used for each overlay. See also Figure 6D.


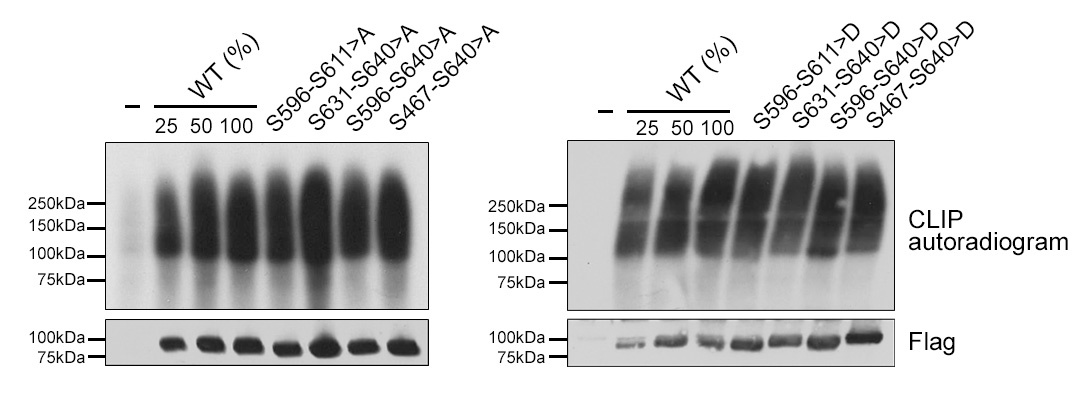


*Figure S5. Phosphosite mutants of Unkempt retain gross RNA-binding capacity.* Binding of the indicated Flag-HA-tagged alanine (>A) (left) or aspartate (>D) (right) mutants of Unkempt or wild-type (WT) Unkempt to RNA in inducible HeLa cells was analyzed by CLIP at 24 hours of treatment with or without (-) doxycycline. Top, CLIP autoradiograms; bottom, immunoblots of the membrane used to develop the autoradiograms. Total alanine and aspartate mutant and (-) samples (100%) were used for the analysis

**Supplemental Materials and Methods**

*Cell culture, immunoprecipitation and western blot analysis*

SH-SY5Y, Neuro-2a (N2a) and HEK293 cells were maintained in Dulbecco’s Modified Eagle’s Medium (DMEM) high glucose (Sigma-Aldrich) supplemented with 10% (v/v) foetal bovine serum (FBS; Sigma-Aldrich) and 1% (w/v) penicillin/streptomycin (Sigma-Aldrich). Cells were trypsinized and sub-cultured twice a week and incubated in 5% CO_2_ at 37^o^C throughout the experiments unless stated otherwise.

For insulin and rapamycin treatment cells were seeded at 7 x 10^5^ cells per well in a six-well plate (Thermo Scientific) in DMEM + 10% FBS, then serum-starved for 16 hours in DMEM alone. Unless stated otherwise, cells were then incubated in DMEM ±1μM insulin (Sigma-Aldrich), or DMEM + 10% FBS ± 1μM rapamycin (LC Laboratories), or another inhibitor, for one hour. 10μM S6K inhibitor (PF4708671, Sigma-Aldrich), 1μM mTOR kinase active site inhibitor (KU0063794, Sigma-Aldrich) and DL001 (Aeovian Pharmaceuticals Inc. at the concentrations shown in Supplemental Figure 1B) were used. Cells were lysed with 0.2% (w/v) SDS (Sigma-Aldrich), 10mM EDTA, 1x protease inhibitor cocktail (Roche), and 1x phosphatase inhibitor cocktail (Sigma-Aldrich) unless stated otherwise.

C57BL/6J mouse primary cortical cells were extracted at E14.5 and one million cells were plated per well in a six well plate in Neurobasal medium (2% (v/v) B27, 2mM glutamine, 0.3% (w/v) glucose, 37.5mM NaCl, 5% (v/v) FBS; ThermoFisher). 48 hours later the media was replaced with 5% (v/v) FBS ± 1μM rapamycin for one hour and lysed as above.

For lambda protein phosphatase treatment cells were serum starved overnight, stimulated with DMEM ± 1μM insulin, then lysed in 50mM Tris pH 7.5, 150mM NaCl, 0.1% (v/v) Triton, 1X protease inhibitor cocktail ± lambda protein phosphatase (New England Biolabs) as per the manufacturer’s instructions and incubated at 30^o^C for one hour.

For glucose and amino acid studies, SH-SY5Y cells were seeded and serum starved as above. This was followed by a 2.5 hour incubation in Hanks Balanced Salt Solution (HBSS, Sigma-Aldrich) to starve cells of amino acids and to reduce the concentration of glucose (to 1g/l) and in some conditions a 30 minute pre-incubation of 1μM rapamycin. Trophic factors were then added to the starved cells for 25 minutes with a combination of glucose, amino acids, FBS (all from Sigma-Aldrich), DMEM no glucose (ThermoFisher), insulin (1μM), or rapamycin (1μM), in continued HBSS or DMEM high glucose medium.

For western blot analysis cell extracts were denatured and reduced in 1x sample buffer (500mM Tris pH 6.8, 40% (v/v) glycerol, 0.2% (w/v) SDS, 2% (v/v) β-mercaptoethanol, and 0.02% (w/v) bromophenol blue) and boiled at 98°C for 10 minutes. Proteins were separated by SDS-polyacrylamide gel electrophoresis (PAGE). Proteins were then transferred onto nitrocellulose membranes (GE Healthcare Life Sciences). Membranes were blocked in 10% fat-free milk powder in tris buffered saline (TBS) (50mM Tris pH 7.4, 150mM NaCl) and probed overnight at 4°C with primary antibodies in 5% (w/v) bovine serum albumen (BSA; Fisher) TBS-T (TBS + 0.1% (v/v) Tween 20). Following four 10 minute washes in TBS-T, membranes were incubated with the appropriate horseradish peroxidase-conjugated secondary antibodies (1:4000) in 5% milk TBS-T for one hour at room temperature. Finally, blots were treated with enhanced chemiluminescence reagents (ECL, GE Healthcare) and imaged using a Kodak or Bio-Rad ChemiDoc system imaging system.

Embryonic brains were lysed in urea lysis buffer (8M urea, 50mM HEPES pH8.2, 10mM glycerol-2-phosphate, 50mM NaF, 5mM sodium pyrophosphate, 1mM EDTA, 1mM sodium orthovanadate, 1mM DTT, cOmplete Protease inhibitor cocktail, Sigma phosphatase inhibitor cocktail III, 500nM okadaic acid) and sonicated briefly. Protein concentration was measured using Pierce BCA Assay kit. Twenty micrograms of brain lysate per embryo were denatured and reduced for 10min at 70^o^C in 1X Pierce LDS sample buffer supplemented with 100mM DTT. Denatured samples were loaded onto pre-cast NuPAGE 4-12%, Bis-Tris protein gel (Invitrogen). Proteins were then transferred onto PVDF membrane using transfer buffer (25mM tris pH8.3, 192mM glycine, 20% v/v methanol) for 1.5hrs at 310mA. Membranes were blocked in 10% w/v skimmed-milk (Marvel) dissolved in TBS-T for 1 hour. Membranes used for immunoblotting with phospho-specific antibodies were washed with TBS-T prior to incubation at 4^o^C overnight with primary antibodies in 5% w/v BSA dissolved in TBS-T. Overnight membranes were then washed three times with TBS-T and incubated with an appropriate HRP-conjugated secondary antibody diluted in 5% w/v milk. After washing the PVDF membranes with TBS-T, they were incubated with enhanced ECL solution (Cytiva) and imaged using Amersham Imager 600 (GE Healthcare). Western blot images were quantified using the Amersham Imager 600 Software.

Primary antibodies used were rabbit anti-Unkempt (1:1000, HPA023636, Cambridge Bioscience), rabbit anti-mTOR (1:1000, #2972, Cell Signalling), rabbit anti-Raptor (1:1000, #2280, Cell Signalling), rabbit anti-β-actin (1:5000, #4967, Cell Signalling), rabbit anti-S6K (1:1000, #9202, Cell Signalling), rabbit anti-phosphoS6K (Thr389, 1:1000, #9234, Cell Signalling), rabbit anti-S6 (1:1000, #2217, Cell Signalling), rabbit anti-phospho-S6 (Ser235/236, 1:1000, #2211, Cell Signalling), rabbit anti-4E-BP1 (1:1000, #9644, Cell Signalling), rabbit anti-phospho-4E-BP1 (Thr37/46, 1:1000, #2855, Cell Signalling), rabbit anti-FLAG (1:2000, F7425, Sigma-Aldrich), rat anti-FLAG (1:2000, #200473-21, Agilent), mouse anti-V5 (1:1000, R960-25, ThermoFisher), mouse anti-HA (1:1000, sc-7392, Santa Cruz), rabbit anti-phosphoUnkempt (1:200, S606, S611, this study), rabbit anti-phospho AKT (Ser473, 1:1000, 4060T, Cell Signalling), rabbit anti-AKT (1:1000, 5691T, Cell Signalling), mouse anti-α-tubulin (1:10000, T9026, clone DM1A, Sigma-Aldrich), rabbit anti-hamartin (TSC1) (1:1000, ab270967, clone EPR24364-109, Abcam), rabbit anti-PKCα (1:1000, ab32376, clone Y124, Abcam), rabbit anti-phospho-PKCα (Ser657, 1:1000, ab180848, clone EPR1901(2), Abcam).

*Expression vectors, cloning, derivation of inducible cell lines, and transfections*

Full length mouse Unkempt was amplified from cDNA using primers 5’-CACCAGATATCCAATGTCGAAGGGCCCCGGGCCCG-3’ and 5’-GACGACTCTAGATCACGACTGGAGGGCATGGGCCC-3’ and cloned into pENTR/D-TOPO (ThermoFisher) according to the manufacturer’s instructions to create pENTR-Unk. Primers used to generate the Unkempt deletions and zinc finger constructs in pENTR-Unk were the same as used previously (1). Primers used to generate the SerL deletion in pENTR-Unk were 5’-GGGCCAGGGGCTGCTGAGCT-3’ and 5’-GCCCACAGGGAGGATGCCCA-3’ and to generate the SerS deletion in pENTR-Unk were 5’-GGGCCAGGGGCTGCTGAGCT-3’ and 5’-GTTATCAAAAGTCTTCTCTAGG-3’. These constructs were then recombined into pcDNA3.1/nV5-DEST (ThermoFisher). pCAG-Rheb^CA^ (also known as pCAG-Rheb^S16H^), was a gift from Angelique Bordey (2).

The alanine and aspartate substitution mutations in Unkempt were created by PCR, using the SuperFi or Phusion Plus mastermix (Thermo Fisher) as per the manufacturer’s instructions using pENTR-Unk as a template. The PCR products were digested using DpnI (NEB) at 37°C for 90 minutes, or where primers were non-overlapping the PCR product was treated with KLD enzyme mix (NEB) for 5 minutes at room temperature, and then transformed into DH5α chemically competent cells. The following primers were used:

S606A

Forward primer - 5’ ACATGGATCTTTGGGTCTG 3’

Reverse primer - 5’ GATGCTGCGGTCCCCAAA 3’

S611A

Forward primer - 5’ GCTTTGGGTCTGAACGGG 3’

Reverse primer - 5’ TCCATGTGATGCTGAGGTC 3’

S606A, S611A:

Forward primer - 5’ GCTTTGGGTCTGAACGGG 3’

Reverse primer - 5’ TCCATGTGATGCTGGGGTC 3’

S565A, S574A

Forward primer – 5’GGCAGCTCAGCTTCCTTCCACGCTGCTTCTCCATCCCCTCCCGTCAGC 3’

Reverse primer – 5’ GCTGAGCTGGCCAGGGCGCCAGGGATGTTCACAGGCGC 3’

S608A, S611A

Forward primer - 5'-TGGGGACCTCAGCAGCACATGGAGCTTTGGGTCTGAAC-3'

Reverse primer - 5'-GTTCAGACCCAAAGCTCCATGTGCTGCTGAGGTCCCCA-3'

S635A, S640A

Forward primer – 5’ GCTTCTCCCCAGGCACTGCCCCTGCCTTCCTAGCAGGGCCAGGGGCTGCTGAG 3’

Reverse primer – 5’ CTCAGCAGCCCCTGGCCCTGCTAGGAAGGCAGGGGCAGTGCCTGGGGAGAAGC 3’

S467-S479>A (S467A, S474A, S475A, S476A, T478A, S479A)

Forward primer – 5’ GACTTCCAGCATCGCTGCCGCTATTGCCGCCAGCTTGGCAGCCACTCCC 3’

Reverse primer – 5’ GCTGGAGGTAATAGCGGCAGCGATGCTGGAAGTCAGGGGGGCGCCCACAGG 3’

S467-S479>D (S467D, S474D, S475D, S476D, T478D, S479D)

Forward primer – 5’ GACTTCCAGCATCGATGACGATATTGACGACAGCTTGGCAGCCACTCCC 3’

Reverse primer – 5’ GCTGTCGTCAATATCGTCATCGATGCGGAAGTCAGGGGGTCGCCCACAGG 3’

T491A

Forward primer – 5’ CTGCAGGCGCCAACAGCACCCCTGGCATGAATGC 3’

Reverse primer – 5’ GCTGTTGGCGCCTGCAGGGCTGGGGGGAG 3’

T491D

Forward primer – 5’ CTGCAGGCGACAACAGCACCCCTGGCATGAATGC 3’

Reverse primer – 5’ GCTGTTGTCGCCTGCAGGGCTGGGGGGAG 3’

S546A

Forward primer – 5’ CACCCCAGCGCCGTCACAATCGGTGGC 3’

Reverse primer – 5’ CGATTGTGACGGCGCTGGGGTGGGGCACTGC 3’

S546D

Forward primer – 5’ CACCCCAGCGACGTCACAATCGGTGGC 3’

Reverse primer – 5’ CGATTGTGACGTCGCTGGGGTGGGGCACTGC 3’

S565-S578>A (S565A, S571A, S574A, S576A, S578A)

Forward primer – 5’ CAGCTCAGCTGCCTTCCACGCTGCTGCTCCAGCCCCTCCCGTCAGCCTCTCC 3’

Reverse primer -

5’ GGGAGGGGCTGGAGCAGCAGCGTGGAAGGCAGCTGAGCTGCCCAGGGCGCCAGGGATG 3’

S565-S578>D (S565D, S571D, S574D, S576D, S578D)

Forward primer – 5’ CAGCTCAGCTGACTTCCACGATGCTGATCCAGACCCTCCCGTCAGCCTCTCC 3’

Reverse primer – 5’ GGGAGGGTCTGGATCAGCATCGTGGAAGTCAGCTGAGCTGCCCAGGTCGCCAGGGAT 3’

S596-S611>A (S596A, S598A, T605A, S606A, S608A, S611A)

Forward primer – 5’ CGTTTTTGGGGGCCGCAGCAGCACATGGAGCTTTGGGTCTGAACGGG 3’

Reverse primer -

5’ CCAAAGCTCCATGTGCTGCTGCGGCCCCCAAAAACGTGTTTTCTGCCTGAGCCAAGTGGCC 3’

S596-S611>D (S596D, S598D, T605D, S606D, S608D, S611D)

Forward primer – 5’ CGTTTTTGGGGGACGACGCAGACCATGGAGATTTGGGTCTGAACGGG 3’

Reverse primer – 5’ CCAAATCTCCATGGTCTGCGTCGTCCCCCAAAAACGTGTTTTCGTCCTGATCCAAGT 3’

S631-S640>A (S631A, T634A, S635A, S640A)

Forward primer – 5’ CCAGGCGCTGCCCCTGCCTTCCTAGCAGGGCCAGGGGCTGC 3’

Reverse primer -

5’ CCCTGCTAGGAAGGCAGGGGCAGCGCCTGGGGCGAAGCTTCCAGAGGC 3’

S631-S640>D (S631D, T634D, S635D, S640D)

Forward primer – 5’ CCAGGCGATGACCCTGCCTTCCTAGACGGGCCAGGGGCTGC 3’

Reverse primer – 5’ CCCGTCTAGGAAGGCAGGGTCATCGCCTGGGTCGAAGCTTCCAGAGGC 3’

Doxycycline-inducible HeLa cells used for the analyses of cellular morphogenesis and for CLIP experiments were generated as described previously (1). Briefly, stably rtTA3-expressing cells were infected ecotropically with retroviruses for TREtight-driven expression of GFP alone (pTt-IGPP) or GFP and either wild-type Unkempt protein or any of the Unkempt alanine or aspartate substitution mutants indicated in Figure 6 and created as described above (pTtight-X-IGPP, where X is either wild-type or mutant Unkempt). To induce transgene expression, double-selected cells were treated with doxycycline (Sigma-Aldrich) at 1 µg/ml.

To generate cells inducibly expressing GFP and either wild-type or S467-S640>A inducible Unkempt and stably expressing Rheb^CA^, HeLa cells were first infected with a lentivirus for UbC promoter-driven expression of Flag-Rheb^CA^, which was produced from the pUltra-puro-RTTA3 vector (Addgene plasmid #58750) in which RTTA3 between BamHI and EcoRI cutting sites was replaced by Flag-Rheb^CA^ amplified from the pCAG-Rheb^CA^ vector. Cells stably expressing Flag-Rheb^CA^ were selected with puromycin and infected with the above viruses to introduce the inducible transgene cassettes.

For transient transfections, HEK293 cells were seeded at 2 x10^5^ cells per well in six well plates (Thermo Fisher) in DMEM/10% FBS. The following day, cells were transfected with V5-tagged Unkempt plasmids at 1 µg/well, using polyethylenimine (Sigma) at 7 µg/well and 200 µl/well of Optimem (Sigma). For co-transfections, Rheb^CA^ was used at 0.5 µg/well and the V5-tagged Unkempt constructs at 1 µg/well. Cells were lysed 48 hours post transfection using 0.2% (w/v) SDS (Sigma-Aldrich), 10mM EDTA, 1x protease inhibitor cocktail (Roche), and 1x phosphatase inhibitor cocktail (Sigma-Aldrich).

To knockdown the expression of Unkempt, SH-SY5Y cells were infected with lentiviruses prepared from pLKO.1-puro vectors expressing either a non-targeting shRNA control (Sigma, SHC016) or human *UNK*-targeting shRNA (targeted sequence: caggtaccaccttcgttacta).

*Overlays of cellular outlines for morphological comparisons*

After 48 hours of incubation with doxycycline, the inducible HeLa cells were

imaged and the outlines of GFP-positive cells were drawn using the Adobe Illustrator software

(Adobe). The outlines of different cells were aligned by the absolute longest cellular process or

the most acuminate part of each outline, and overlaid by superimposing the cell bodies, as done previously (1).

*Co-immunoprecipitation experiments and liquid chromatography with tandem mass spectrometry (LC-MS/MS) analysis*

To induce overexpression of and immunoprecipitate FLAG-HA-Unkempt, previously described HeLa S3 cells stably expressing doxycycline-inducible FLAG-HA-Unkempt (1) were used. They were cultured in DMEM (high glucose; Sigma) supplemented with 10% (v/v) FBS (Sigma) and 1% penicillin/streptomycin (Lonza) in 15 cm dishes (Nunc, ThermoFisher) to 90% confluency and induced with doxycycline hyclate (Sigma-Aldrich) at 1 µg/ml for 24 hours. Non-induced cells were also cultured in parallel to use as a negative control. 16 hours prior to co-immunoprecipitation, cells were serum starved in DMEM, in the presence of doxycycline. 10% FBS was then re-introduced to culture media and cells were treated with 1 μM rapamycin (LC Laboratories) or DMSO for one hour. Dishes were then washed with ice-cold PBS and cells were scraped and lysed on ice for 15 minutes in 3 ml lysis buffer.

For co-immunoprecipitations, one 15 cm dish was used for each condition and cells were lysed in either CHAPS lysis buffer (120 mM NaCl, 40 mM HEPES pH 7.5, 1 mM EDTA, 0.3% (w/v) CHAPS, 1X protease inhibitor cocktail, 1X phosphatase inhibitor cocktail and 0.2 mM PMSF) or Triton X-100 lysis buffer (120 mM NaCl, 40 mM HEPES pH 7.5, 1 mM EDTA, 1% Triton X-100, 1X protease inhibitor cocktail, 1X phosphatase inhibitor cocktail and 0.2 mM PMSF). Samples were then centrifuged at 17,000 x g for 10 minutes at 4^o^C. The supernatant was transferred to a new tube and protein concentration was determined using Protein Assay Dye Reagent Concentrate (Bio-Rad) following the manufacturer’s instructions. Samples were diluted and standardized to the condition with lowest protein concentration (≤ 2 mg/ml) using their respective lysis buffer. Equal volumes of diluted lysates were added to pre-washed 50 μl anti-FLAG M2 affinity agarose gel slurry (Sigma-Aldrich) and incubated rotating end over end for two hours at 4^o^C. The agarose beads were then washed three times with lysis buffer followed by three additional washes with wash buffer (150 mM NaCl, 50 mM HEPES pH 7.5). Excess buffer was removed using an insulin needle (30G) and beads were resuspended in 60 µl 2 x sample buffer and protein was eluted by heating at 95^o^C for 5 min. Co-immunoprecipitants were resolved on an 8% SDS-PAGE gel followed by protein transfer on nitrocellulose membrane (GE Healthcare Life Sciences). Western blot analysis was performed as described above.

For LC-MS/MS analysis of immunoprecipitated Unkempt, cells were cultured in two 15 cm dishes for each condition and samples were processed in a similar way to co-immunoprecipitation experiments but using a different lysis buffer (25 mM Tris pH 8.0, 150 mM NaCl, 5% (v/v) glycerol, 1% Triton (v/v) X-100, 1X protease inhibitor cocktail, 1X phosphatase inhibitor cocktail and 0.2 mM PMSF) and wash buffer (50 mM Tris pH 8.0, 150 mM NaCl). Sample dilution and standardization prior to incubation with anti-FLAG resin was omitted. Bound Unkempt was eluted with 40 µl 2x sample buffer heated at 95^o^C for 5 minutes. 35μl of each eluate was resolved on a 4-12% Bis-Tris Novex NuPAGE gel (ThermoFisher) for coomassie staining and mass spec analysis. The remaining 5 µl were resolved on the same gel and used for FLAG western blotting to confirm the immunoprecipitation and exact molecular weight of Unkempt. Half of the gel for LC-MS/MS analysis was fixed (7% (v/v) glacial acetic acid, 40% (v/v) methanol, 53% HPLC-grade water) for 30 minute shaking at room temperature. The fixed gel was stained overnight at room temperature (4 parts 1X Brilliant Blue-G Colloidal Concentrate (Sigma-Aldrich) and 1-part methanol, freshly mixed). The gel was destained three to four times (5% (v/v) glacial acetic acid, 25% (v/v) methanol, 70% HPLC-grade water) until background was significantly reduced and bands were clearly visible. Bands of the expected FLAG-HA-Unkempt size (~90 kDa) for both conditions, rapamycin and DMSO, were excised using sterile scalpel in a fume hood and stored in HPLC-grade water.

LC-MS/MS analysis was performed by the Cambridge Centre for Proteomics, University of Cambridge. Gel fragments were transferred into a 96-well PCR plate. The bands were cut into 1mm^2^ pieces, destained with a solution 50% acetonitrile and 50mM ammonium bicarbonate, reduced with 10mM DTT for one hour at 37^o^C and alkylated with 55mM iodoacetamide at room temperature in the dark for 45 minutes, then subjected to enzymatic digestion with 0.01µg/µl sequencing grade chymotrypsin (Promega V1061) overnight at 37°C. After digestion, the supernatant was pipetted into a sample vial and loaded onto an autosampler for automated LC-MS/MS analysis.

All LC-MS/MS experiments were performed using a Dionex Ultimate 3000 RSLC nanoUPLC (Thermo Fisher Scientific Inc, Waltham, MA, USA) system and a QExactive Orbitrap mass spectrometer (Thermo Fisher Scientific Inc, Waltham, MA, USA). Separation of peptides was performed by reverse-phase chromatography at a flow rate of 300 nL/minute and a Thermo Scientific reverse-phase nano Easy-spray column (Thermo Scientific PepMap C18, 2 µm particle size, 100A pore size, 75 µm i.d. x 50cm length). Peptides were loaded onto a pre-column (Thermo Scientific PepMap 100 C18, 5 µm particle size, 100A pore size, 300 µm i.d. x 5mm length) from the Ultimate 3000 autosampler with 0.1% (v/v) formic acid for three minutes at a flow rate of 10 µL/minute. After this period, the column valve was switched to allow elution of peptides from the pre-column onto the analytical column. Solvent A was water + 0.1% (v/v) formic acid and solvent B was 80% (v/v) acetonitrile, 20% water + 0.1% (v/v) formic acid. The linear gradient employed was 2-40% B in 30 minutes.

The LC eluant was sprayed into the mass spectrometer by means of an Easy-Spray source (Thermo Fisher Scientific Inc.). All *m/z* values of eluting ions were measured in an Orbitrap mass analyzer, set at a resolution of 70000 and was scanned between *m/z* 380-1500. Data dependent scans (Top 20) were employed to automatically isolate and generate fragment ions by higher energy collisional dissociation (HCD, NCE:25%) in the HCD collision cell and measurement of the resulting fragment ions was performed in the Orbitrap analyser, set at a resolution of 17500. Singly charged ions and ions with unassigned charge states were excluded from being selected for MS/MS and a dynamic exclusion window of 20 seconds was employed.

Post-run, the data was processed using Protein Discoverer (version 2.1., ThermoFisher). Briefly, all MS/MS data were converted to mgf files and the files were then submitted to the Mascot search algorithm (Matrix Science, London UK) and searched against the UniProt_Mus_musculus_20180514 database (61295 sequences; 27622875 residues). The peptide and fragment mass tolerances were set to 20 ppm and 0.1 Da, respectively. A significance threshold value of p<0.05 and a peptide cut-off score of 20 were also applied. Following Mascot analysis, data were converted into .sf3 file and sent to user for further analysis.

Converted files were analysed using Scaffold4 Proteome Software (version 4.8.7). Protein threshold was set to 95% with minimum number of peptides at 2. Peptide threshold was relaxed and each phospho-peptide was examined manually accounting for accurate identification of amino acids, number of peptides with the same phosphorylation site and peptide identification probability percentage. All selected sites were less than 10 delta ppm.

The mass spectrometry proteomics data have been deposited to the ProteomeXchange Consortium via the PRIDE (3) partner repository with the dataset identifier PXD034469 and 10.6019/PXD034469.

Prediction disorder probability was performed using the online tool Protein DisOrder prediction System (PrDOS, <http://prdos.hgc.jp/cgi-bin/top.cgi>) (4) applying 5% prediction false positive rate. Mouse Unkempt protein sequence was used. Protein sequences were aligned using Clustal Omega.

*In vitro mTORC1-Rheb kinase assays*

HeLa S3 cells stably expressing doxycycline inducible FLAG-HA-Unkempt were used (1). After serum starvation, stimulation and rapamycin treatment, as described above, cells from three 15 cm dishes per condition were scraped and pooled together. They were lysed for 20 min on ice in 10 ml lysis buffer (25 mM Tris pH 8.0, 150 mM NaCl, 5% (v/v) glycerol, 1% (v/v) Triton X-100, 1X protease inhibitor cocktail, 1X phosphatase inhibitor cocktail and 0.2 mM PMSF). Lysates were centrifuged at 20,000 x g for 20 minutes at 4^o^C and the cleared supernatant was incubated with 70 µl anti-FLAG M2 affinity agarose gel rotating end over end overnight at 4^o^C. Samples were then washed twice in lysis buffer followed by three washes in high salt buffer (50 mM Tris pH 8.0, 500 mM NaCl, 5% (v/v) glycerol, 1% (v/v) Triton X-100) and three times in wash buffer (50 mM Tris pH 8.0, 150 mM NaCl). Recombinant FLAG-HA-Unkempt was eluted in 80 µl wash buffer supplemented with 0.2 mg/ml 3 x FLAG peptide (Generon) rotating end over end for 30 min at 4^o^C. Purified Unkempt was collected using 30G needle, flash-frozen in liquid nitrogen and stored at -80^o^C until required.

Radiolabelled *in vitro* mTORC1 kinase assays with Rheb-GTP were performed as described previously (5) using FLAG-HA-Unkempt purified as described above.

For the *in vitro* kinase assays for LC-MS/MS analysis, HEK293 cells grown in twelve 10 cm dishes were co-transfected with active myc-mTOR E2914K mutant (Addgene) and HA-Raptor (6), each at 9 µg per plate. In parallel another eight 10 cm dishes of HEK293 cells were transfected with 18 µg of the constitutively active mutant GST-FLAG-Rheb Q64L (Rheb^CA^) (7). 48 hours later cells co-transfected with mTOR and Raptor were serum starved overnight and then stimulated with 100 nM insulin (Sigma-Aldrich) for 15 minutes. Cells were washed in ice-cold PBS and each plate was lysed in 1 ml mTOR lysis buffer (40 mM HEPES pH 7.4, 50 mM NaCl, 2 mM EDTA, 10 mM B-glycerophosphate, 0.3% (w/v) CHAPS) on ice for 10 minutes. Samples were centrifuged for 10 minutes at 17,000 x g at 4^o^C. Cleared supernatants were pooled together and incubated with 3 µl anti-c-Myc antibody (9E10, DSHB) per 1 ml lysate, rotating end over end for 1.5 hours at 4^o^C. The lysate was divided equally and incubated with 50 µl of prewashed 50% Protein G slurry (GE Healthcare) for each condition, rotating for one hour at 4^o^C. Beads were then washed once with low salt mTOR wash buffer (40mM HEPES pH 7.4, 150mM NaCl, 2mM EDTA, 10mM B-glycerophosphate, 0.3% (w/v) CHAPS), twice with high salt mTOR wash buffer (40mM HEPES pH 7.4, 400mM NaCl, 2mM EDTA, 10mM B-glycerophosphate, 0.3% (w/v) CHAPS), and twice with HEPES/KCl wash buffer (25mM HEPES pH 7.4, 20mM KCl). Cells transfected with GST-FLAG-Rheb Q64L were washed in ice-cold PBS and each plate was lysed in 1 ml Rheb lysis buffer (40 mM HEPES pH 7.4, 50 mM NaCl, 10 mM pyrophosphate, 10 mM glycerophosphate, 5 mM MgCl_2_, 0.3% (w/v) CHAPS, 1X protease inhibitor cocktail) for 10 minutes on ice followed by centrifugation for 10 minutes at 17,000 xg at 4^o^C. Supernatants were incubated with 30 µl prewashed Glutathione sepharose 4B slurry (GE Healthcare) per 1 ml lysate, rotating end over end for two hours at 4^o^C. Beads were then washed twice with Rheb lysis buffer and once with Rheb storage buffer (20 mM HEPES pH 8.0, 200 mM NaCl, and 5 mM MgCl_2_). The supernatant was removed with 30G needle and bound GST-Rheb was eluted in 40 µl Rheb elution buffer (20 mM HEPES pH 8.0, 200 mM NaCl, and 5 mM MgCl_2_, 10 mM reduced Glutathione). To prepare the mTORC1 complex inhibitor, 5 µg FKBP12 was incubated in 50 µl of 30 mM rapamycin in 1x kinase assay buffer (25mM HEPES pH 7.4, 20mM KCl, 10mM MgCl_2_) for five minutes in the dark at room temperature. For each condition, mTOR/Raptor immunoprecipitates (bound on beads) were incubated with 50 µl 3x kinase assay buffer, 25 µl GST-FLAG-Rheb Q64L, with or without 10 µl FKBP12:rapamycin complex, made up to 150 µl total reaction volume and incubated on ice for 20 minutes. The *in vitro* kinase assay was started by adding 50 µl assay start buffer (25mM HEPES pH 7.4, 10mM MgCl_2_, 140mM KCl, 500uM ATP) and 1 µg purified FLAG-HA-Unkempt, or recombinant GST-4E-BP1 (5) as a positive control. Samples were incubated for one hour shaking at 30^o^C. The reaction was stopped with 4 x sample loading buffer and stored at -20^o^C. For mass spectrometry analysis, kinase assay reaction samples were diluted in 1 x kinase assay buffer/assay start buffer (3:1) and concentrated using Amicon Ultra-0.5 centrifugal filter unit with a 3 kDa cutoff. Concentrated samples were processed and analysed by LC-MS/MS as described above. Positive control samples were used for western blotting to confirm mTORC1 activity as assessed by phosphorylation of 4EBP-1. Primary antibodies used were mouse anti-c-Myc (1:1000, 9E10, DSHB), mouse anti-HA (1:1000, SC-7392, Santa Cruz), rat anti-FLAG (1:2000, #200473-21, Agilent), rabbit anti-4E-BP1 (1:1000, #9644, Cell Signalling), rabbit anti-phospho-4E-BP1 (Thr37/46, 1:1000, #2855, Cell Signalling).

*UV-crosslinking and immunoprecipitation (CLIP) assays*

CLIP experiments were carried out essentially as described in (8).

GFP or GFP and Unkempt-inducible HeLa cells were grown in 10 cm plates and

harvested at 80% confluency. Prior to harvest, the inducible HeLa cells were treated with doxycycline for 24 hours to induce transgene expression. Cells were washed, irradiated with UV-light (254 nm) at 150 mJ/cm^2^ on ice, scraped into three 2 ml tubes, and spun down at 4°C. The supernatant was removed and the cell pellets were frozen at -80°C until use. Immunoprecipitation of the cross-linked Unkempt-RNA complexes was carried out using anti-Flag antibody (F1804, Sigma-Aldrich). Samples were processed exactly as described in (8) until the autoradiography stage where the membrane was exposed to X-ray film (the 3’ end dephosphorylation and first adapter ligation steps were omitted). The experiment was performed in three independent biological replicates. All replicates yielded comparable results.

**Supplemental References**

1. Murn, J., Zarnack, K., Yang, Y. J., Durak, O., Murphy, E. A., Cheloufi, S., Gonzalez, D. M., Teplova, M., Curk, T., Zuber, J., Patel, D. J., Ule, J., Luscombe, N. M., Tsai, L. H., Walsh, C. A., and Shi, Y. (2015) Control of a neuronal morphology program by an RNA-binding zinc finger protein, Unkempt. *Genes Dev* **29**, 501-512

2. Hartman, N. W., Lin, T. V., Zhang, L., Paquelet, G. E., Feliciano, D. M., and Bordey, A. (2013) mTORC1 targets the translational repressor 4E-BP2, but not S6 kinase 1/2, to regulate neural stem cell self-renewal in vivo. *Cell reports* **5**, 433-444

3. Perez-Riverol, Y., Bai, J., Bandla, C., García-Seisdedos, D., Hewapathirana, S., Kamatchinathan, S., Kundu, D. J., Prakash, A., Frericks-Zipper, A., Eisenacher, M., Walzer, M., Wang, S., Brazma, A., and Vizcaíno, J. A. (2022) The PRIDE database resources in 2022: a hub for mass spectrometry-based proteomics evidences. *Nucleic Acids Res* **50**, D543-d552

4. Ishida, T., and Kinoshita, K. (2007) PrDOS: prediction of disordered protein regions from amino acid sequence. *Nucleic Acids Res* **35**, W460-464

5. Dunlop, E. A., Dodd, K. M., Seymour, L. A., and Tee, A. R. (2009) Mammalian target of rapamycin complex 1-mediated phosphorylation of eukaryotic initiation factor 4E-binding protein 1 requires multiple protein-protein interactions for substrate recognition. *Cell Signal* **21**, 1073-1084

6. Kim, D. H., Sarbassov, D. D., Ali, S. M., King, J. E., Latek, R. R., Erdjument-Bromage, H., Tempst, P., and Sabatini, D. M. (2002) mTOR interacts with raptor to form a nutrient-sensitive complex that signals to the cell growth machinery. *Cell* **110**, 163-175

7. Long, X., Lin, Y., Ortiz-Vega, S., Yonezawa, K., and Avruch, J. (2005) Rheb binds and regulates the mTOR kinase. *Curr Biol* **15**, 702-713

8. Buchbender, A., Mutter, H., Sutandy, F. X. R., Körtel, N., Hänel, H., Busch, A., Ebersberger, S., and König, J. (2020) Improved library preparation with the new iCLIP2 protocol. *Methods (San Diego, Calif.)* **178**, 33-48
